# The current status of heavy metal in lake sediments from China: Pollution and ecological risk assessment

**DOI:** 10.1002/ece3.3124

**Published:** 2017-06-12

**Authors:** Yongfeng Xu, Yi Wu, Jiangang Han, Pingping Li

**Affiliations:** ^1^ Co‐Innovation Center for the Sustainable Forestry in Southern China Nanjing China; ^2^ College of Biology and the Environment Nanjing Forestry University Nanjing China

**Keywords:** China, ecological risk assessment, heavy metal, lake sediment, pollution

## Abstract

Heavy metal contamination in lake sediments is a serious problem, particularly in developing countries such as China. To evaluate heavy metal pollution and risk of contamination in lake sediments on a national scale in China, we collated available data in the literature of the last 10 years on lake sediments polluted with heavy metals from 24 provinces in China. Based on these data, we used sediment quality guidelines, geoaccumulation index, and potential ecological risk index to assess potential ecological risk levels. The results showed that approximately 20.6% of the lakes studied exceeded grade II level in Chinese soil quality standards for As, 31.3% for Cd, 4.6% for Cu, 20.8% for Ni, 2.8% for Zn, and 11.1% for Hg, respectively. Besides, the mean concentrations for As in 10.3% of lakes, Hg in 11.9% of lakes, and Ni in 31.3% of lakes surpassed the probable effect level. The potential ecological risk for toxic metals decreased in the order of Cd > Hg > As > Cu > Pb > Ni > Cr > Zn, and there were 21.8% of the lakes studied in the state of moderate risk, 10.9% in high risk, and 12.7% in very high risk. It can be concluded that Chinese lake sediments are polluted by heavy metals to varying degrees. In order to provide key management targets for relevant administrative agencies, based on the results of the pollution and ecological risk assessments, Cd, Hg, As, Cu, and Ni were selected as the priority control heavy metals, and the eastern coastal provinces and Hunan province were selected as the priority control provinces. This article, therefore, provides a comprehensive assessment of heavy metal pollution in lake sediments in China, while providing a reference for the development of lake sediment quality standards.

## 1 INTRODUCTION

There are a great number of lakes in China, with a total area of 81,415 km^2^, accounting for 0.9% of the country's land. Lakes are an important multifunctional surface water resource and play a key role in water supply, flood control, irrigation, aquaculture, climate regulation, and the maintenance of ecological balance (Guo, Huo, Xi, Zhang, & Wu, 2015; Hansen, 2012; Le et al., 2010; Ra, Bang, Lee, Kim, & Kim, 2011). Sediments are an important part of the water body, located at the junction of the solid–liquid interface, and have a significant influence on the structure and function of wetland ecosystems (Chen et al., 2016). The contamination of lake sediments by heavy metals has become one of the hot topics in the field of environmental science because of their potential biological toxicity, environmental durability, and biological accumulation (Jiang, Wang, Wang, Zhang, & Hu, 2012; Reddy, Basha, Kumar, Joshi, & Ramachandraiah, 2004; Varol, 2011).

Sediments can adsorb heavy metals in water and in turn reduce the level of pollution of the water. However, the pollutants can be released from the sediments when environmental conditions such as electrical conductivity (EC), pH, temperature, sediment particle size, oxidation–reduction potential change in the water or in the sediments and can cause secondary pollution to the water environment (Dong et al., 2014; Eggleton & Thomas, 2004; Fu et al., 2014; Ndimele, 2012; Zhang, Juying, Mamat, & Qing, 2016). Sediments are also the main habitat and food source of benthic organisms, and sediment pollution is detrimental to aquatic organisms directly or indirectly and may have even further adverse impact on terrestrial organisms and human beings as a result of bioaccumulation (Järup, 2003; Kaushik, Kansal, Santosh, Kumari, & Kaushik, 2009; Segura, Arancibia, Zúñiga, & Pastén, 2006). For example, Tao, Yuan, Xiaona, and Wei (2012) reported that Pb was the most bioconcentrated element in certain aquatic organisms, which may damage the human nervous, skeletal, and immune systems in cases of excessive intake of the metal through aquatic products (Bryan & Langston, 1992). The excessive intake of Hg can harm both the renal and nervous systems, while the excessive intake of As and Cd has adverse effects on skin, blood vessels, nervous system, and lung, kidney, prostate, bone, respectively (Al‐Saleh et al., 2014; Żukowska & Biziuk, 2008). Therefore, it is necessary to investigate heavy metal pollution in lake sediments and assess ecological risks caused by heavy metals in order to protect the corresponding aquatic ecological environment and human health.

In China, due to rapid urbanization and industrialization, heavy metal contamination in lake sediment has become a very serious problem (Cheng et al., 2015; Guo et al., 2015). A great number of studies reported that many Chinese lakes have been seriously polluted by heavy metals and the potential threats are growing (Cheng et al., 2015; Guo et al., 2015; Jiang et al., 2012; Li et al., 2013; Wen, Shan, & Zhang, 2012). Recently, heavy metal pollution in lake sediment has drawn much attention from many researchers because of the lack of effective supervision and management (Guo et al., 2015; Yuan, Shen, Liu, Wang, & Meng, 2011). Heavy metal contamination in lakes varies with local economic development, pollution sources, and geographical conditions (Cheng et al., 2015; Thevenon et al., 2013; Zan et al., 2012). Generally, exogenous inputs of heavy metals from human activities and rapid economic development are the main sources of heavy metals in lake sediments (Zhang et al., 2016). For example, the total volume of wastewater discharged inadequately increases year by year and reached 71.6 billion tons in 2015 (National Bureau of Statistics, 2015), which may cause metals to discharge into lakes. Heavy metals in concentrations beyond food safety standards have appeared in aquatic products that are still sold as edible foods (He, Jiang, Dai, & Li, 2013; Wang, Xu, Sun, Liu, & Li, 2013).

A review of the literature shows that a great number of studies of heavy metal pollution in lake sediments have been carried out in China during the last 10 years. However, most previous studies were small scale, reporting on an individual lake or a small number of lakes, but no significant work has been carried out on a regional or larger scale. This study assesses the pollution levels and ecological risks of lake sediment on a national scale in China, to help compare heavy metal concentrations in lake sediments from different regions and analyze the pollution sources and their controls under the constraint of the same risk assessment method.

The main goals of this study were (1) to evaluate the heavy metal pollution levels of lake sediments in China; (2) to assess the ecological risks posed by these contaminated lake sediments; (3) and to propose solutions for the environmental management of polluted lakes in China.

## 2 MATERIALS AND METHODS

### 2.1 Data collection and processing

We systematically collected studies related to heavy metal pollution from lake sediments in China from the past 10 years. According to the priority heavy metal pollutants designation by the USEPA (Li, Ma, Kuijp, Yuan, & Huang, 2014), eight heavy metals, namely As, Cd, Cr, Cu, Ni, Pb, Zn, and Hg, were selected. According to recent surveys, there are a great number of lakes in China, including 2693 lakes with areas larger than 1 km^2^, 581 lakes with areas larger than 10 km^2^ and 127 lakes with areas larger than 100 km^2^ (Ma et al., 2011), many of which are in different degrees of eutrophication (Ding et al., 2015). The data from 110 lakes located in 24 provinces throughout China were collected and analyzed, from the main literature databases including Web of Science and China Knowledge Full‐text Literature Database. Sixty‐eight datasets on the As content, 103 datasets on the Cd content, 107 datasets on the Cr content, 108 datasets on the Cu content, 96 datasets on the Ni content, 109 datasets on the Pb content, 107 datasets on the Zn content, and 45 datasets on the Hg content of heavy metals in lake sediments were collated (Table S1). The basic characteristics of these lakes are also shown in Table S1. The area of almost all these lakes is more than 10 km^2^, making up a large proportion of the total lake area in their respective provinces. In addition, many of the lakes in this study are a source of drinking water and have varying degrees of eutrophication, making them the current research hot spots in China (Wu et al., 2008). For example, Poyang Lake in Jiangxi province and Qinghai Lake in Qinghai province are the largest freshwater and saltwater lake in China, respectively (Yuan et al., 2011; Zhu, Chen, & Fu, 2013); Taihu Lake in Jiangsu province, Chaohu Lake in Anhui province, and Dianchi Lake in Yunnan province are the most serious polluted lakes in China (Jiang et al., 2012; Wang, Yao, Liu, & Liu, 2014; Wen et al., 2012); and Suyahu Lake in Henan province is the largest artificial lake in China (and in Asia); all are severely affected by heavy metal contamination (Ma et al., 2016; Zhang et al., 2013). Therefore, to a certain extent, each province is representative of the varying degrees of lake contamination.

The sampling strategies and processing methods used in these experimental studies are summarized in Table S2. The lake sediment samples were mainly collected to a depth of 0–5 cm, 0–10 cm, 0–20 cm and then mixed thoroughly to give a composite sample (Table S2). In general, they were air‐dried at room temperature and then sieved and pulverized. The sieved lake sediment samples were complete digested with a mixed acid such as HF, HCLO_4_, HNO_3_, HCL, Aqua regia or H_2_SO_4_ (Table S2). Finally, the total concentrations of eight heavy metals were determined by ICP, ICP‐AES, ICP‐OES, ICP‐MS, AAS, AFS, XFS, XRF, or CVAFS (Table S2). These lake sediment sampling strategies and processing methods are all widely accepted by the scientific community.

**Figure 1:**
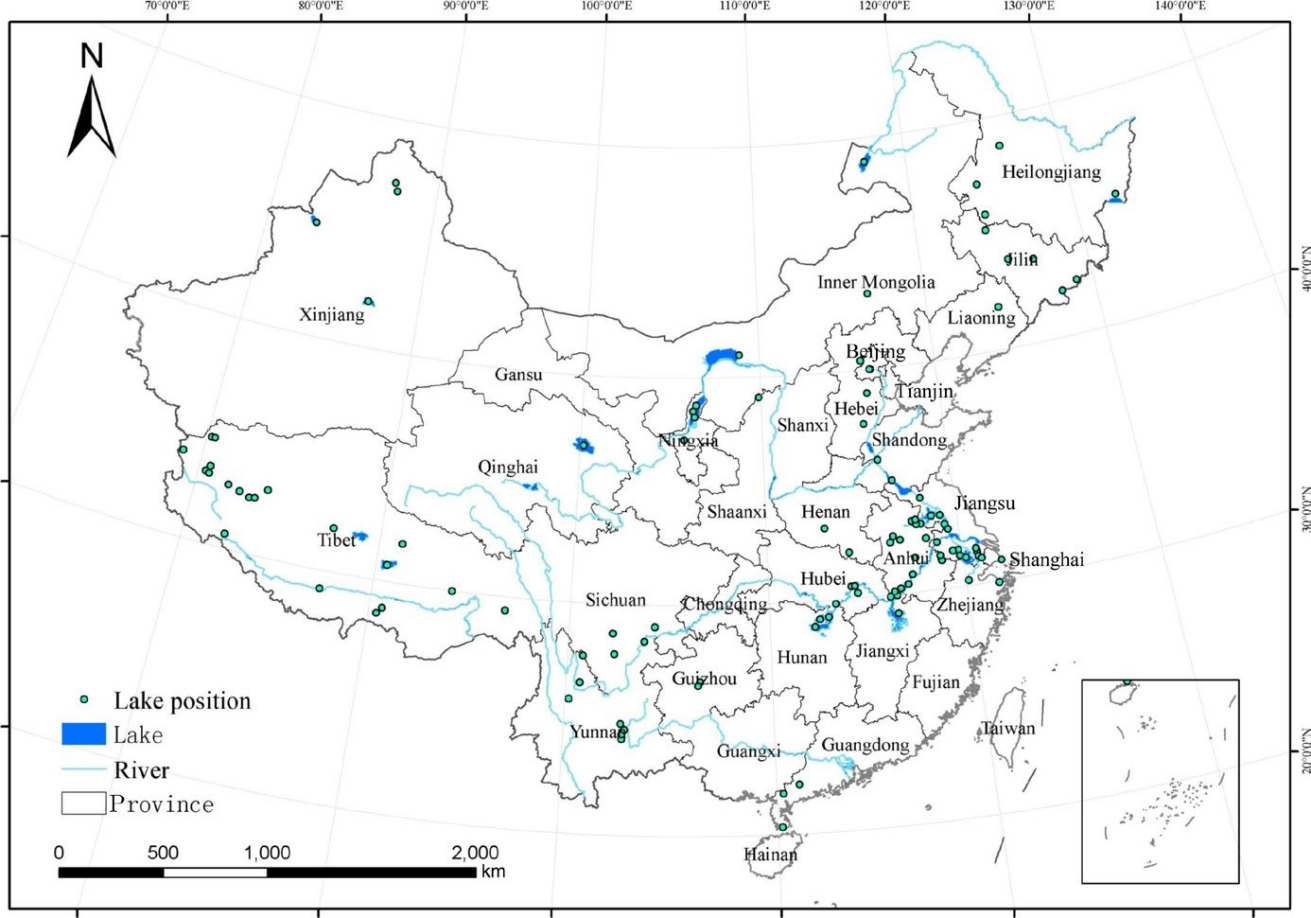
Distribution map of the 110 researched lakes in China (green dots)

**Figure 2:**
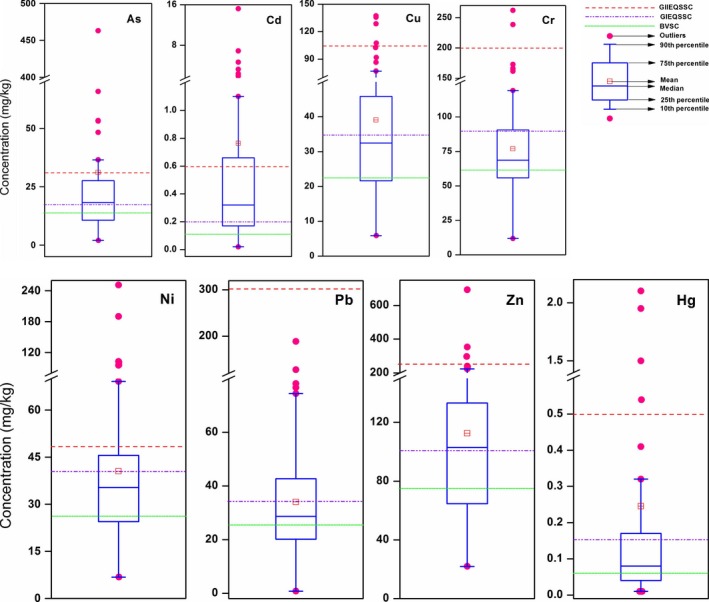
Boxplots of the heavy metal concentration (mg/kg) in sediment from collected lakes. The mean and median heavy metal (As: *n *=* *68; Cd: *n *=* *103; Cr: *n *=* *107; Cu: *n *=* *108; Ni: *n *=* *96; Pb: *n *=* *109; Zn: *n *=* *107; Hg: *n *=* *45) concentration are compared with GIEQSSC (purple dotted line), GIIEQSSC (red dotted line), and BVSC (green solid line). GIEQSSC: grade I environment quality standard for soils in China; GIIEQSSC: grade II environment quality standard for soils in China; BVSC: Background values for soils in China

The distribution map of the lakes included in this analysis is presented in Figure 1. In order to corroborate the comparisons with standards, percentile values (10th, 25th, 50th, 75th, and 90th), grades I and II environment quality standard for soils in China, background values for soils in China, and mean concentration of the heavy metal are presented (Figure 2 and Table S3). All the calculations, the charts, and statistical analyses for the data were performed with EXCEL 2003 and ORIGIN 8.5.

### 2.2 Ecological risk assessment

At present, there are many methods to evaluate the ecological risk of heavy metals, including the method of geoaccumulation index (*I*_geo_) (Muller, 1969; Sutherland, 2000), enrichment factors (Sutherland, 2000; Tania, Micaela, & Malcolm, 2004), sediment quality guidelines (SQGs) (Long, MacDonald, Smith, & Calder, 1995; Spencer & Macleod, 2002), SEM/AVS (Chai, Li, & Zan, 2017; Prica, Dalmacija, Rončević, Krčmar, & Bečelić, 2008), pollution load index (Chakravarty & Patgiri, 2009; Long, Field, & MacDonald, 1998), potential ecological risk index (Hakanson, 1980; Suresh, Sutharsan, Ramasamy, & Venkatachalapathy, 2012), nemerow pollution index (Yan et al., 2016), and so on. In order to get comprehensive and accurate evaluation results, we combined the method of SQGs, I_geo_, and potential ecological risk index to assess the ecological risk of heavy metal pollution in sediments.

#### 2.2.1 Sediment quality guidelines

The SQGs method was introduced by MacDonald, Ingersoll, & Berger, (2000) and has been used to evaluate the sediment quality and detect the degree of contamination of an aquatic ecosystem. It can also be directly applied to a new area, particularly where local SQGs are unavailable (Cheng et al., 2015; Xu, Cao, Zhang, Li, & Hu, 2016). SQGs consist of the comparable threshold effects level (TEL) and probable effects level (PEL) (Table 1), which can be used to determine whether a specific metal detected in sediment poses a threat to aquatic ecosystems. Adverse biological effects are not expected to occur when values are below the TEL. Adverse biological effects can occasionally occur when values are between the TEL and the PEL, while adverse biological effects occur more frequently when they exceed the PEL.

**Table 1:** Comparisons of the concentrations of heavy metals in Chinese lake sediment with other studies, TEL and PEL (mg/kg)

Item	As	Cd	Cr	Cu	Ni	Pb	Zn	Hg
TEL^a^	7.2	0.68	52.3	18.7	15.9	30.2	124	0.174
PEL^a^	41.6	4.21	160.4	108.2	42.8	112.2	271	0.486
China^b^ (110 lakes; Mean)	31.17	0.76	76.98	39.09	40.54	34.08	112.62	0.25
China^c^ (72 mining soils; Mean)	195.5	11.0	84.28	211.9	106.6	641.3	1163	3.82
China^d^ (38,393 soils; Mean)	12.1	0.225	68.5	27.1	29.6	31.2	79.0	0.087

NA, not available.

a MacDonald et al. (2000).

b This paper.

c Li et al. (2014).

d Chen et al. (2015).

#### 2.2.2 Index of geoaccumulation

The *I*_geo_ was introduced by Muller (1969) and can not only be used to assess whether or not sediments have been contaminated by heavy metals (Tiwari, Sahu, Bhangare, Ajmal, & Pandit, 2013), but can also more effectively reflect sediment quality (Chaudhary, Ahmad, Mashiatullah, Ahmad, & Ghaffar, 2013). The *I*_geo_ for the lake sediments was calculated using the following equation:

(1) $$I_{\mathrm{geo}}={\text{log}}_{2}\left(C_{n}/1.5B_{n}\right),$$

where *C*_*n*_ is the measured concentration of every heavy metal identified in the lake sediments (mg/kg), and *B*_*n*_ is the geochemical background value, collected from the natural geochemical background concentrations of heavy metals at the A soil layer (0–20 cm) in every province in China (CNEMC, 1990) (Table S4). The factor of 1.5 was used for correction of regional background differences. According to the value of *I*_geo_, contamination can be classified in seven grades and the classes of 0–6 are as follows: practically uncontaminated (*I*_geo_ ≤ 0), uncontaminated to moderately contaminated (0 < *I*_geo_ ≤ 1), moderately contaminated (1 < *I*_geo_ ≤ 2), moderately to heavily contaminated (2 < *I*_geo_ ≤ 3), heavily contaminated (3 < *I*_geo_ ≤ 4), heavily to extremely contaminated (4 < *I*_geo_ ≤ 5), or extremely contaminated (*I*_geo_ > 5) (Muller, 1969). The *I*_geo_ values of heavy metals in sediments from different lakes are listed in Table S5.

#### 2.2.3 Index of potential ecological risk

The potential ecological risk index was introduced by Hakanson (1980) and can not only be used to assess the degree of toxic metal pollution in sediments but also indicate the degree of biological risk (Yan et al., 2013; Yi, Yang, & Zhang, 2011). This index can be calculated as follows:

(2) $$\text{RI}=\sum_{i=1}^{n}E_{r}^{i}=\sum_{i=1}^{n}\left(T_{r}^{i}\times C^{i}/C_{n}^{i}\right),$$

where RI is computed as the sum of all potential ecological risk indices for heavy metals in sediment from lakes. $E_{r}^{i}$ is the potential ecological risk index of single heavy metal *i* in sediment from lakes. $T_{r}^{i}$ is the toxicity response factor for heavy metal *i*, where $T_{r}^{i}$ for As, Cd, Cr, Cu, Ni, Pb, Zn, and Hg are 10, 30, 2, 5, 2, 5, 1, and 40, respectively (Hakanson, 1980). *C* ^*i*^ is the measured concentration of heavy metal *i* and $C_{n}^{i}$ is the reference value of heavy mental *i* collected from the natural geochemical background concentrations of heavy metals at the A soil layer (0–20 cm) in every province in China (CNEMC, 1990) (Table S4). According to the value of $E_{r}^{i}$ and RI, 5 grades of $E_{r}^{i}$ and 4 grades of RI can be classified as low risk ($E_{r}^{i}$ ≤ 40), moderate risk (40 < $E_{r}^{i}$ ≤ 80), high risk (80 < $E_{r}^{i}$ ≤ 160), very high risk (160 < $E_{r}^{i}$ ≤ 320), or extremely high risk ($E_{r}^{i}$ > 320); and low risk (RI* *≤ 150), moderate risk (150 < RI* *≤ 300), high risk (300 < RI ≤ 600), or very high risk (RI > 600), respectively. The $E_{r}^{i}$ and RI values of heavy metals in sediments from different lakes and the proportion of grades of potential ecological risk in whole lake from China are listed in Tables S6 and S7, respectively.

## 3 RESULTS

### 3.1 Overview of heavy metal concentrations in lake sediments

As shown in Figure 2, the concentration of heavy metals in lake sediments in China has a wide range, and the mean and median concentrations of each heavy metal both exceed the corresponding BVSC (background values for soils in China). The mean concentrations of As, Cd, Cr, Cu, Ni, Pb, Zn, and Hg are approximately 2.1, 3.9, 0.9, 1.1, 1.0, 1.0, 1.1, and 1.6 times greater than the grade I level and 1.0, 1.3, 0.4, 0.4, 0.8, 0.1, 0.5, and 0.5 times greater than grade II level in Chinese soil quality standard, respectively (GB 15618‐1995). The exceedance rate of lake sediments for each heavy metal decreased in the order of Cd (70.9%) > Zn (56.1%) > As (52.9%) > Cu (44.4%) > Pb (39.4%) > Ni (38.5%) > Hg (33.3%) > Cr (26.2%) in comparison with the grade I level in Chinese soil quality and in the order of Cd (31.3%) > Ni (20.8%) > As (20.6%) > Hg (11.1%) > Cu (4.6%) > Zn (2.8%) > Cr (1.9%) > Pb (0.0%) in comparison with the grade II level in Chinese soil quality, respectively (Table 2a). From this analysis, the lake sediments in China are contaminated the most by Cd and the least by Cr.

**Table 2:** Percentages of heavy metals in each item (As: *n* = 68; Cd: *n* = 103; Cr: *n* = 107; Cu: *n* = 108; Ni: *n* = 96; Pb: *n* = 109; Zn: *n* = 107; Hg: *n* = 45)

Item	As	Cd	Cr	Cu	Ni	Pb	Zn	Hg
(a) Compared with environment quality standard for soils in China	Exceeding rate of lake sample in each standard (%)							
Grade I level	52.9	70.9	26.2	44.4	38.5	39.4	56.1	33.3
Grade II level	20.6	31.1	1.9	4.6	20.8	0.0	2.8	11.1
(b) Compared with TEL and PEL	% of lake sample in each guideline							
<TEL	8.8	74.8	20.6	14.8	7.3	48.6	69.2	75.6
≥TEL < PEL	80.9	22.3	73.8	80.6	59.4	50.5	28.0	13.3
≥PEL	10.3	2.9	5.6	4.6	33.3	0.9	2.8	11.1
(c) The class of *I*_geo_	% of lake sample in each class							
Practically uncontaminated (0)	60.3	20.4	85.0	49.1	71.9	72.5	56.1	51.2
Uncontaminated to moderately contaminated (1)	32.4	22.3	13.1	45.3	25.0	26.6	39.3	28.9
Moderately contaminated (2)	2.9	30.1	1.9	3.7	2.1	0.9	2.8	8.9
Moderately to heavily contaminated (3)	2.9	12.6	0.0	1.9	0.0	0.0	0.9	4.4
Heavily contaminated (4)	1.5	8.7	0.0	0.0	1.0	0.0	0.9	2.2
Heavily to extremely contaminated (5)	0.0	2.9	0.0	0.0	0.0	0.0	0.0	0.0
Extremely contaminated (6)	0.0	2.9	0.0	0.0	0.0	0.0	0.0	4.4
(d) The grade of ecological risk	% of lake sample in each grade							
Low risk	95.5	16.5	100.0	99.1	100.0	100.0	100.0	39.9
Moderate risk	1.5	19.4	0.0	0.9	0.0	0.0	0.0	17.8
High risk	1.5	35.0	0.0	0.0	0.0	0.0	0.0	26.7
Very high risk	1.5	15.5	0.0	0.0	0.0	0.0	0.0	6.7
Extremely high risk	0.0	13.6	0.0	0.0	0.0	0.0	0.0	8.9

The corresponding values of heavy metals based on SQGs and compared with other studies are listed in Table 1. According to the TEL and PEL values of heavy metals in lake sediment, the mean concentrations of seven heavy metals (As, Cd, Cr, Cu, Ni, Pb, and Hg) were between the corresponding TEL and PEL except that of Zn (Table 1). As shown in Table 2b, more than 80% of As, Cr, Cu, and Ni concentrations were higher than the corresponding values of TEL, suggesting that adverse biological effects are expected to occur (MacDonald et al., 2000). The mean concentrations for As in 10.3% of lakes, Hg in 11.1% of lakes, and Ni in 31.3% of lakes surpass the corresponding values of PEL (Table 2b). Thus adverse biological effects occur more frequently due to the concentrations of As, Hg, and Ni.

A comparison of heavy metal data of Chinese lake sediments with published data of Chinese soils and mining soils are shown in Table 1. The mean concentration of all heavy metals in the 110 lakes studied is lower than that of the mining soils and about 1–3 times that of the soils. From this analysis, it indicated that the degree of pollution by heavy metals in Chinese lake sediments is relatively severe, especially for As and Cd.

### 3.2 Pollution assessment of researched lake sediment metals using *I*_geo_

**Figure 3:**
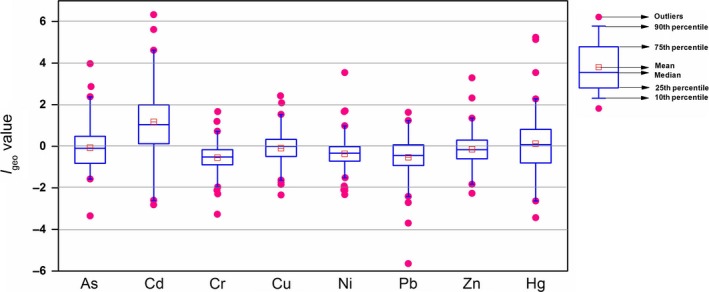
Boxplots of *I*_geo_ values for heavy metals (As: *n *=* *68; Cd: *n *=* *103; Cr: *n *=* *107; Cu: *n *=* *108; Ni: *n *=* *96; Pb: *n *=* *109; Zn: *n *=* *107; Hg: *n *=* *45) in Chinese lake sediments

Figure 3 displays the boxplots of the *I*_geo_ values for heavy metals in Chinese lake sediments. Table 2c lists the proportion of class distribution for pollution assessment of heavy metals using the *I*_geo_ in lakes in China. As shown in Figure 3 and Table 2c, the *I*_geo_ values indicate that all the sediments in lakes for Cr and Pb fall below class 2, with 85.0% and 72.5% falling into class 0, respectively. For As, Ni, and Zn, more than 90% of the *I*_geo_ values in lakes are lower than class 1 (Table 2c). For Cu, the *I*_geo_ values vary from class 0 to class 3, with 94.4% falling below class 1. However, the Cd and Hg *I*_geo_ values vary the most, ranging from class 0 to class 6, and more than 50% of the *I*_geo_ values lie above class 2 for Cd, while approximately 50% of the *I*_geo_ values is above class 1 for Hg.

As shown in Figure 3, the average *I*_geo_ value for Cr, As, Pb, Ni, and Zn are −0.46, −0.35, −0.22, −0.21, and −0.16, respectively, placing them into the class of practically uncontaminated. Average *I*_geo_ values for Hg and Cu in sediment from lakes lie between 0 and 1, indicating that the lakes can be classed as uncontaminated to moderately contaminated. The average Cd *I*_geo_ value is 1.64, suggesting moderately contaminated level. All things considered it can be concluded that the contamination levels of the eight heavy metals decreased generally in the order of Cd > Hg > Cu > Zn > Ni > Pb > As > Cr (Figure 3).

**Figure 4:**
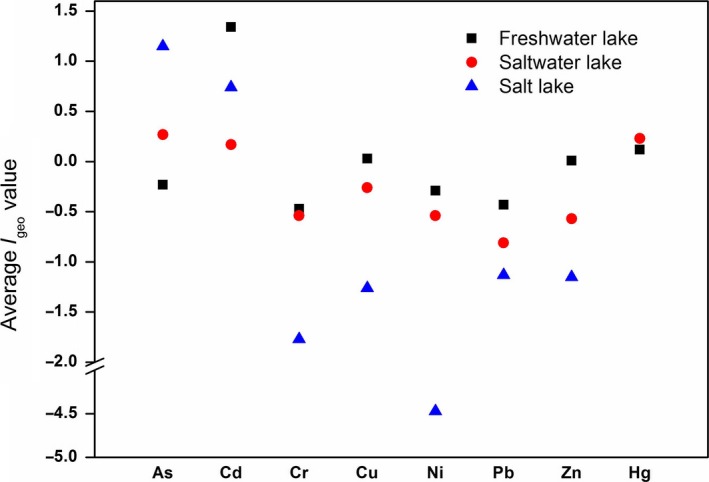
Average *I*_geo_ values of heavy metals for different types of researched lakes. Different shapes represent different types of lakes. The squares represent lakes from Freshwater Lake. The circles indicate lakes from Saltwater lake. Lakes from Salt lake are marked as triangles

Lake type, human activities, economic development, pollution history, and distance to emission sources may affect lake pollution to various degrees (Guo, Liu, Zhang, Hou, & Zhang, 2016; Guo et al., 2015). Figure 4 shows the average *I*_geo_ values of heavy metals for different types of lakes. As can be seen, among the national lake sediments, average *I*_geo_ values for Cr, Cu, Ni, Pb, and Zn in saltwater lake sediment were below 0, which can be labeled as practically uncontaminated. Freshwater lake sediment showed the highest *I*_geo_ values for Cd, Cr, Cu, Ni, Pb, and Zn, while saltwater lake sediment showed the highest *I*_geo_ values for As and freshwater lake sediment showed the highest *I*_geo_ values for Hg. In addition, it is worth noting that the average *I*_geo_ values for Cd and Hg from freshwater lake and saltwater lake were higher than 0, suggesting that the lake sediments in China have been contaminated by Cd and Hg because of human activities (Muller, 1969).

According to calculation results (Table 3), for the 24 provinces from which lakes were sampled, Beijing, Hebei, Hunan, Jilin, Liaoning, Zhejiang, and Guangdong provinces are the most polluted provinces with at least one heavy metal between 4 and 6 on the *I*_geo_ scale or containing two or more heavy metals of at least 3 on the *I*_geo_ scale. Besides, the *I*_geo_ values for Cd in Jiangsu and Sichuan provinces are 2.58 and 2.76, respectively. This indicates that the lake sediments in these provinces are moderately to heavily contaminated by Cd. The other provinces with *I*_geo_ values of less than 2 for all heavy metals appear to be the least polluted. In addition, the highest *I*_geo_ values for Cd (5.29) were found in Hunan province, while the highest *I*_geo_ values for Hg (5.13) were found in Liaoning province. This indicates that the lake sediments in these provinces are the most heavily polluted by the corresponding metals.

**Table 3:** Average *I*_geo_ values of the researched lake sediments by province in China

Province	As	Cd	Cr	Cu	Ni	Pb	Zn	Hg
Anhui	0.09	1.40	−0.28	0.38	−0.06	0.04	0.33	1.50
Beijing		3.26	−0.38	−0.70	−0.14	−0.37	−0.26	−0.79
Guangdong		3.12	0.58	1.17	2.18	0.46	2.39	1.55
Guizhou	−0.01	−0.20	−0.90	1.02	1.70	−0.48	0.13	2.00
Heilongjiang	−0.96	0.01	−0.52	−0.58	−0.39	−0.66	−0.71	−0.47
Hebei	−0.99	4.62	−0.74	−0.08	−0.68	0.24	−0.60	0.74
Henan	−0.80	1.23	−0.61	−0.27	−0.41	−0.38	−0.14	−2.35
Hubei	0.17	1.11	−0.29	0.19	−0.21	−0.08	0.35	0.47
Hunan	0.28	5.29	−0.18	0.44		0.25	0.08	0.05
Inner Mongolia	−0.03	0.82	−0.32	0.05	−0.37	−0.32	−0.45	0
Jiangsu	0.29	2.58	−0.80	0.16	−0.25	−0.50	0.24	−1.64
Jiangxi	−0.89	0.57	−0.13	−0.14	−0.12	−0.20	−0.06	−0.66
Jilin	−0.86	2.55	−0.40	1.03	0.52	−0.52	0.54	3.72
Liaoning		3.88	0.17	1.38	0.51	0.47	1.07	5.13
Ningxia	−0.82	0.25	−0.54	−0.51	−0.81	−0.47	−0.40	−0.07
Qinghai	−0.96	−0.19	−0.70	−0.49	−1.30	−0.41	−0.48	
Shanghai	−0.78	−0.28	−0.72	−0.84	−1.13	−0.27	−0.20	0.25
Shaanxi	−0.71	0.27	−0.75	−0.74		−0.70	−1.09	−1.17
Sichuan	−0.84	2.76	−0.02	0.54	0.27	0.13	0.13	−0.87
Shandong	0.62	1.05	−0.18	0.31	0.02	−0.27	−0.02	1.07
Tibet	1.37	1.15	−0.59	−0.28	−0.40	−0.56	−0.49	
Xinjiang	−0.11	−0.47	−0.60	−0.60	−0.64	−0.31	−0.61	0.23
Yunnan	−0.47	1.18	0.19	0.13	−0.14	−0.37	−0.02	0.71
Zhejiang	−0.09	2.76	−0.41	−0.11		0.28	−0.26	2.58

### 3.3 Potential ecological risk assessment of lake sediment metals and risk mapping

Table 2d summarizes the proportion of grades of ecological risk of single heavy metals, and Table S7 describes the proportion of grades of potential ecological risk in Chinese lakes. The potential ecological risk indices of Cr, Ni, Pb, and Zn in all studied lakes were lower than 40, which suggests slight potential ecological risk of the corresponding metals in all studied lakes. For As, the percentage of low risk, moderate risk, high risk, and very high risk accounted for 95.5%, 1.5%, 1.5%, and 1.5%, while the percentage of low risk and moderate risk accounted for 99.1% and 0.9% for Cu, respectively (Table 2d). The $E_{r}^{i}$ values of Cd and Hg vary the most; however, more than 50% of the studied lakes are in the state between high risk and extremely high risk for Cd, while more than 50% of the lakes are in the state between low risk and moderate risk for Hg. For metals, RI risk estimation in lakes was found to be low risk, moderate risk, high risk, and very high risk, accounting for 54.6%, 21.8%, 10.9%, and 12.7% in studied lakes, respectively (Table S7).

**Figure 5:**
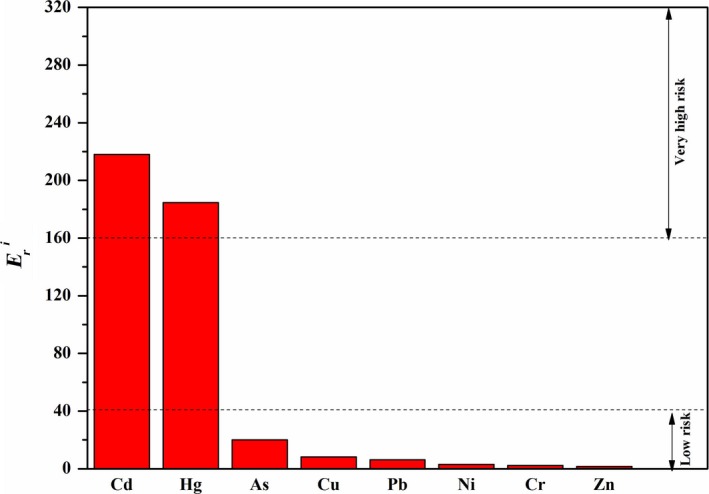
Comparison of the potential ecological risk index ($E_{r}^{i}$) for different metals in lake sediments on a national scale. The $E_{r}^{i}$ values of 0–40 are classified as low risk, and the $E_{r}^{i}$ values of 160–320 are classified as Very high risk

From Figure 5, the potential ecological risk for toxic metals decreased in the order of Cd (218.1) > Hg (184.6) > As (20.1) > Cu (8.3) > Pb (6.3) > Ni (3.0) > Cr (2.3) > Zn (1.6). Average potential ecological risk index ($E_{r}^{i}$) and potential toxicity response index (RI) values of the lake sediments by province are listed in Table 4. The $E_{r}^{i}$ values of As, Cr, Cu, Ni, Pb, and Zn are lower than 40 in all provinces, which is classed as a low degree of potential ecological risk. For Cd, the $E_{r}^{i}$ values in Beijing, Guangdong, Hebei, Hunan, and Liaoning provinces appear to pose an extremely high risk, with $E_{r}^{i}$ values higher than 320. Besides, the $E_{r}^{i}$ values for Hg in the provinces of Zhejiang (358.1), Jilin (789.2), and Liaoning (2108.1) are also found to be at an extremely high risk level (Table 4).

**Table 4:** Average $E_{r}^{i}$ and RI values of the researched lake sediments by province in China

Province	$E_{r}^{i}$								RI
	As	Cd	Cr	Cu	Ni	Pb	Zn	Hg	
Anhui	15.9	118.8	2.5	9.7	2.9	7.7	1.9	169.7	329.1
Beijing		429.7	2.3	4.6	2.7	5.8	1.3	34.8	481.2
Guangdong		391.1	4.5	16.9	13.6	10.3	7.9	176.1	620.3
Guizhou	14.9	39.2	1.6	15.2	9.7	5.4	1.6	240.0	327.6
Heilongjiang	7.7	45.3	2.1	5.0	2.3	4.7	0.9	43.2	111.3
Hebei	7.6	1,104.3	1.8	7.1	1.9	8.9	1.0	100.0	1,232.4
Henan	8.6	105.4	2.0	6.2	2.3	5.8	1.4	11.8	143.3
Hubei	16.9	97.1	2.5	8.6	2.6	7.1	1.9	78.3	215.0
Hunan	18.2	1,764.3	2.7	10.2		8.9	1.6	62.1	1,867.9
Inner Mongolia	14.7	79.2	2.4	7.8	2.3	6.0	1.1	60.0	173.5
Jiangsu	18.3	269.5	1.7	8.4	2.5	3.5	1.5	19.3	326.8
Jiangxi	8.1	66.7	2.7	6.8	2.8	6.5	1.4	38.1	133.2
Jilin	8.3	263.6	2.3	15.3	4.3	5.2	2.2	789.2	1,090.3
Liaoning		661.1	3.4	19.5	4.3	10.4	3.2	2,108.1	2,809.8
Ningxia	8.5	53.6	2.1	5.3	1.7	5.4	1.1	57.1	134.8
Qinghai	7.7	39.4	1.8	5.3	1.2	5.6	1.1		62.3
Shanghai	8.7	37.0	1.8	4.2	1.4	6.2	1.3	71.6	132.1
Shaanxi	9.2	54.3	1.8	4.5		4.6	0.7	26.7	101.7
Sichuan	8.4	303.8	3.0	10.9	3.6	8.2	1.6	32.8	372.3
Shandong	23.1	92.9	2.6	9.3	3.0	6.2	1.5	126.3	265.0
Tibet	38.7	100.0	2.0	6.2	2.3	5.1	1.1		155.3
Xinjiang	13.9	32.5	2.0	4.9	1.9	6.0	1.0	70.6	132.9
Yunnan	10.8	102.3	3.4	8.2	2.7	5.8	1.5	98.3	233.0
Zhejiang	14.1	304.3	2.3	7.0		9.1	1.3	358.1	696.1

**Figure 6:**
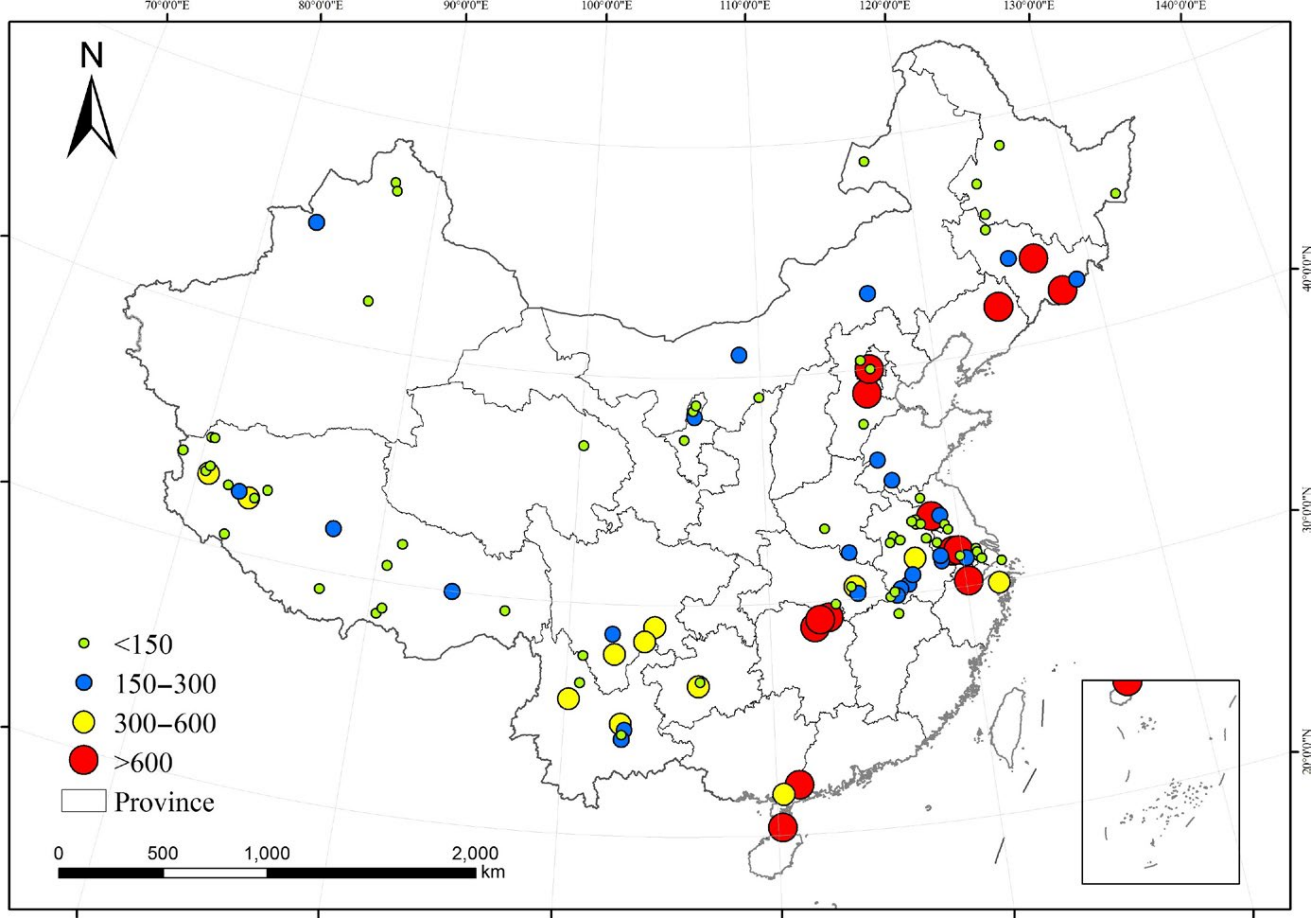
Distribution of potential ecological risk indices (RI) of lake sediment heavy metals in China. Different colors represent different potential ecological risk levels. The green dots represent low risk (RI ≤ 150). The blue dots indicate moderate risk (150 < RI ≤ 300). High risk (300 < RI ≤ 600) and very high risk (RI > 600) are marked as yellow and red dots, respectively

Figure 6 shows the distribution of potential ecological risk indices of each lake sediment heavy metals in China. Figure 6 reveals the number of lakes exceeding extremely high risk values defined by national/regional standards. There are three in Hunan and Jiangsu province each, two in Guangdong and Jilin province, and one in Beijing, Hebei, Liaoning, and Zhejiang province. From Table 4, for metals in the whole of the lakes, RI risk estimation was found to be “very high” in Hebei, Hunan, Jilin, Liaoning, and Zhejiang provinces (RI ≥ 600); “high” in Anhui, Beijing, Guangdong, Guizhou, Jiangsu, and Sichuan provinces (300 ≤ RI < 600); “moderate” in Hubei, Inner Mongolia, Shandong, Tibet, and Yunnan provinces (150 ≤ RI < 300); and “low” in the remaining provinces (RI < 150).

### 3.4 The relationship between pollution and economic development

**Figure 7:**
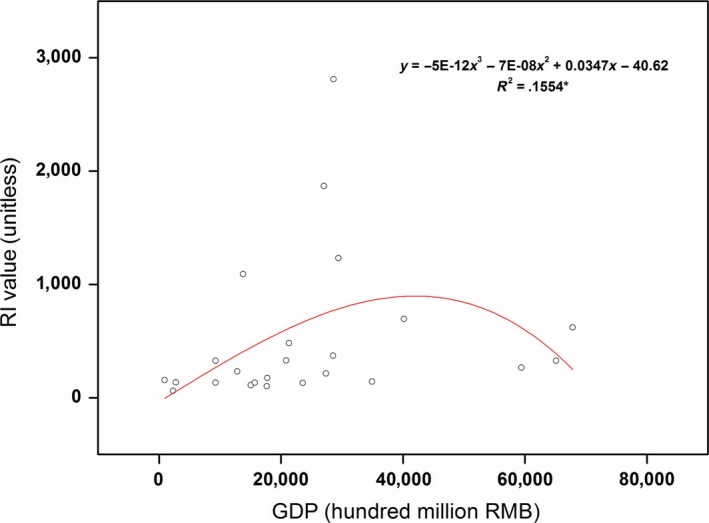
The model ($y=b_{0}+b_{1}x+b_{2}x^{2}+b_{3}x^{3}$) (Alam et al., 2016) was used to fit the relationship between lake sediment pollution (RI values: *y*) and economic development (GDP values in 2014: *x*) (*n *=* *24). * indicates significant at 5% level

Several studies have stated that the concentrations of heavy metals discharge may be consistent with the local economic development level (Guo et al., 2015; Hu & Cheng, 2013). In order to evaluate the relationship between the pollution in lake sediment and economic development in 24 provinces, we used the gross domestic product (GDP) for different provinces derived from the National Bureau of Statistics of China (National Bureau of Statistics, 2015). The model ($y=b_{0}+b_{1}x+b_{2}x^{2}+b_{3}x^{3}$) (Alam, Murad, Noman, & Ozturk, 2016) was used to fit the relationship between lake sediment pollution (RI values) (*y*) and economic development (GDP values) (*x*) (Fig. 7). Figure 7 shows that the lake sediment pollution in each province is significantly correlated (*p < *.05) with GDP, the fitting curve presenting like a “reverse U.” The results indicate that the risk of pollution in lake sediments increases with the development of economy, but decreases when a turning point is reached.

## 4 DISCUSSION

According to the data collected on heavy metal concentrations in lake sediments, it can be concluded that lake sediments are contaminated nationwide by heavy metals to varying degrees. Relatively speaking, As, Cd, Cr, Cu, Ni, and Hg are the comparatively highest pollutants. China is one of the largest global producers and consumers of metals and metalloids (Chen, Teng, Lu, Wang, & Wang, 2015), suggesting that the rapidly growing anthropogenic activities may lead to more heavy metal inputs to lakes because of rapid economic development. In particular, since the policy of reform and opening to the outside world, the rapid development of urbanization and industrialization in China has drastically increased industrial and municipal wastewater discharges, causing greatly increasing concentrations of heavy metals in lake sediments and other environment media (Fan & Luo, 2013; Hu & Cheng, 2013). Based on the pollution assessment above, approximately 31.3%, 20.8%, and 20.6% of lakes had levels of Cd, Ni, and As exceeding their corresponding grade II level in Chinese soil quality standard, respectively. Additionally, adverse biological effects occur more frequently due to the concentrations of As, Hg, and Ni based on SQGs. Therefore, Cd, As, Hg, and Ni were selected as priority control heavy metals. According to the *I*_geo_ assessment, the average *I*_geo_ values for eight heavy metals decreased generally in the order of Cd > Hg > Cu > 0 >  Zn > Ni > Pb > As > Cr. Similarly, Guo et al. (2015) inferred that for heavy metals in typical lakes in China, Cr, Pb, and Ni appeared to cause the least contamination, while Cd and Cu presented the higher *I*_geo_ values for most studied lakes. Similar results were shown in mining soils (Li et al., 2014), urban soils, and agricultural soils (Wei & Yang, 2010). Thus, Cd, Hg, and Cu were also selected as priority control heavy metals. Moreover, based on the potential ecological risk index assessment, lake sediments have been polluted nationally at a very high risk level by Cd and Hg, with other metals presenting a low degree of potential ecological risk. This ranking is not surprising, as it is likely associated with large emissions of Cd, Hg, and As from human activities in China (Cheng et al., 2015). Cheng et al. (2014) reported that the total emissions of Cd are estimated at about 743.77 tons in 2009, of which the contributions of industrial processes and combustion sources are approximately 56.6% and 43.4%, respectively. The total emissions of Cd from wastewater discharge were 17.25 tons in 2014 (National Bureau of Statistics, 2015), while the total emissions of Cd from coal combustion have rapidly increased from 31.14 tons in 1980 to 261.52 tons in 2008, at an annual average growth rate of 8.0% (Tian et al., 2012). In addition, Tian et al. (2010) estimated that the national emissions of Hg and As from coal burning quickly increased from 73.59 tons and 635.57 tons in 1980 to 305.95 tons and 2,205.50 tons in 2007, at an annually averaged growth rate of 5.4% and 4.7%, respectively. Therefore, emissions from anthropogenic sources have resulted in these metals depositing and accumulating in lake sediments through atmospheric deposition or surface runoff. Altogether, based on the above analyses, Cd, Hg, As, Cu, and Ni were selected as priority control heavy metals in Chinese lake sediments.

Based on the previous assessment, for the 24 provinces in which the lake sediments were studied, Beijing, Hebei, Hunan, Jilin, Liaoning, Zhejiang, and Guangdong provinces appear to be the most significantly polluted provinces. Similar results were found in previous research by Guo et al. (2015), who stated that lake sediment pollution was higher in the Eastern Plain Region and Northeast China Region due to dense population and economic development. However, because of the limited number of studied lakes in some provinces, further authentication of these results is needed. According to the potential ecological risk index assessment, the risk values in Beijing, Guangdong, Hebei, Hunan, and Liaoning provinces are higher. Emissions of Cd from heavy metals in industrial wastewater are mainly concentrated in Hunan, Guangxi, Jiangxi, Hubei, and Fujian, while the emissions of Hg are mainly concentrated in Hunan, Guangxi, Guangdong, Liaoning, and Gansu provinces, accounting for more than 90% of their total emissions (Fan & Luo, 2013). Besides, Tian et al. (2012) reported that emissions of Cd, Cr, and Pb are highly concentrated in the northern and eastern region provinces, such as Hebei, Shanxi, and Shandong provinces, driven by dramatic coal consumption by the industrial and power plant sectors. Overall, the spatial pattern indicates that lake sediment pollution in eastern coastal areas of China is relatively higher than that in western provinces, which may be related to increasing human activities and economic development in eastern regions. According to previous studies, emissions of Hg, Cd, Pb, and As from industrial wastewater in 2003–2010 were mainly concentrated in southern provinces (e.g., Hunan province) and eastern coastal provinces (e.g., Zhejiang and Jiangsu provinces) (Fan & Luo, 2013), and emission of Cd, Cr, and Pb from coal combustion in 2008 were highly concentrated in provinces of northern and eastern regions (e.g., Shandong, Hebei, and Shaanxi provinces) (Tian et al., 2012). This implies that the heavy metal contamination in lake sediment in eastern coastal areas may be related to high wastewater discharge and energy production. In addition, due to mineral exploitation, heavy metal pollution is concentrated in southern provinces as well as in the developed eastern coastal areas, whereas low pollution levels exist in west and northwest China (Li et al., 2014). As a result, the eastern coastal provinces and Hunan province were selected as priority control provinces.

At present, China faces the challenging task of balancing economic development with the protection of lake environments. As mentioned before, heavy metals in lake sediments are derived from both natural and anthropogenic sources. Among them, primary anthropogenic sources include wastewater discharge, energy production (e.g., oil and coal burning), and mineral exploitation (Fan & Luo, 2013; Li et al., 2014; Tian et al., 2012). Therefore, relevant administrative agencies must take measures to alleviate the pressure of environmental pollution in lakes. The most effective way to reduce heavy metal pollution in lakes is efficient control of the pollution sources and strict enforcement of environmental regulations, especially in terms of wastewater discharge (Chen, Zheng, Tu, & Zhu, 1999). So, it is necessary to control pollutant discharge from different sources including sewage sludge, urban sewage, mine soil, industrial, agriculture, and aquaculture wastewater. Based on analyses presented in this article, freshwater lakes should also be given more attention. With increasing industrial and municipal wastewater discharges, more and more sewage is discharged directly into lakes without being treated and may easily accumulate in lake sediments. According to the National Bureau of statistics of China (National Bureau of Statistics, 2015), over 70,000 million tons of wastewater have been discharged in China, among which the emissions of Cd and Hg were more than 17,000 and 740 kg, respectively. Due to such high amounts of Cd and Hg inputs into lakes, the safety of aquatic products, particularly of fish, should be paid close attention to. Chinese fisheries have stood as the world's top producer for many years, with a total production of 43.5 million tons, accounting for 61.7% in the world food fish aquaculture production in 2013 (He, 2015; He et al., 2016). Additionally, fish is a major component of the diet of residents around the lakes because of their age‐old aquaculture tradition and the advantageous geographical situation (He et al., 2016). At the same time, restored ecosystems along the lakeshore can effectively intercept pollutants from the process of migration. Several studies have reported that willow can effectively absorb Cd, Cr, Cu, Ni, Pb, and Zn, while other trees such as *Acer pseudoplatanus* L., *Alnus glutinosa* L. Gaertn., *Fraxinus excelsior* L., *Populus alba* L., and *Robinia pseudoacacia* L. can also reduce the risk of metal dispersal (Meers, Vandecasteele, Ruttens, Vangronsveld, & Tack, 2007; Mertens, Vervaeke, De Schrijver, & Luyssaert, 2004). In order to reduce the release of pollutants from lake sediments, aquatic plants such as *Scirpus maritimus* and *Juncus maritimus* can also be used (Almeida, Mucha, & Vasconcelos, 2006; Peng, Song, Yuan, Cui, & Qiu, 2009). In addition, in order to promote economic growth and address environmental problems, we should avoid reaching a turning point and look for a suitable model of economic development (Figure 7). Effective measures such as converting energy sources to natural gas, developing both traditional and high‐tech clean coal technology, establishing mechanisms of strategic oil reserve, developing fuel cell and hydrogen vehicles, promoting desulfurization, improving environmental monitoring and management, among others, should be considered (Alam et al., 2016).

## 5 CONCLUSION

Through a systematic assessment using SQGs, *I*_geo_ and potential ecological risk index methods, this study analyzes data from 110 Chinese lakes in 24 provinces and gives the first description of the overall pollution status of heavy metals in Chinese lake sediments and risks posed to the ecological environment. According to our pollution assessment, it is apparent that the Chinese lake sediments are polluted by heavy metals to varying degrees. Lake sediments have been contaminated the most by Cd. In order to reduce hazards of heavy metal pollution and protect the ecological environment surrounding lakes, special attention should be paid to Cd, Hg, As, Cu, and Ni which have been selected as the priority control heavy metals. In addition, the eastern coastal provinces and Hunan province have been identified as priority control provinces for heavy metal pollution of lake sediments.

## CONFLICT OF INTEREST

None declared.
