# Supplementary material for: The current status of heavy metal in lake sediments from China: Pollution and ecological risk assessment
Source: Ecol Evol. 2017 Jun 12;7(14):5454–66. doi: 10.1002/ece3.3124 (PMC5528247; doi:10.1002/ece3.3124)
Supplement: Supplementary file 1 [file ECE3-7-5454-s001.doc]

**Appendix S. -- The current status of heavy metal in lake sediments from China: Pollution and ecological risk assessment**

Yongfeng Xu ^1,2^, Yi Wu ^2^, Jiangang Han ^2*^, Pingping Li ^1*^

^1^ Co-Innovation Center for the Sustainable Forestry in Southern China, Nanjing 210037, China

^2^ College of Biology and the Environment, Nanjing Forestry University, Nanjing 210037, China

^*^ Corresponding authors at: College of Biology and the Environment, Nanjing Forestry University, Nanjing 210037, China, Fax:+86-25-85427668 (J. Han); Co-Innovation Center for the Sustainable Forestry in Southern China, Nanjing 210037, China, Fax:+86-25-85427255 (P. Li)

E-mail addresses: [hanjiangang76@126.com](mailto:zhuyongli76@126.com (Y. Zhu)；hanjiangang76@126.com) (J. Han); lipingping@ujs.edu.cn (P. Li)

**Supplementary Materials:**

Table S1 The concentrations of each heavy metal of sediments in single lake and basic characteristics.

Table S2 Materials and methods of heavy metal contamination in sediment from lakes in China.

References for Table S1 and S2

Table S3 The concentrations (mg/kg) for the eight heavy metals from the researched lake sediments in China.(BVSC: Background values for soils in China; GIEQSSC: Grade I environmental quality standard for soils in China; GIIEQSSC: Grade II environmental quality standard for soils in China)

Table S4 Background concentration values (mg/kg) of heavy metals in the soils of certain provinces in China

Table S5 I_geo_ values of heavy metals in sediments from different lakes in China.

Table S6 $E_{r}^{i}$ and RI values of heavy metals in sediments from different lakes in China.

Table S7 The proportion of grades of potential ecological risk in whole lake from China.

**Table S1 The concentrations of each heavy metal of sediments in single lake and basic characteristics.**

| Province | Number | Lake (Classification by the degree of mineralization) | Area (km^2^) | Average depth (m) | Concentration (mg/kg) | | | | | | | | Reference |
| --- | --- | --- | --- | --- | --- | --- | --- | --- | --- | --- | --- | --- | --- |
|  |  |  |  |  | As | Cd | Cr | Cu | Ni | Pb | Zn | Hg |  |
| Anhui | L1 | Chaohu Lake(Freshwater) | 774.0 | 4.09 | 20.2 | 0.43 | 56.9 | 26.2 | 33.1 | 49.8 | 153.7 | 0.24 | Yin et al., 2011; Hua et al., 2006 |
|  | L2 | Longganhu Lake(Freshwater) | 223.2 | 3.78 |  | 0.37 | 85.4 | 50.7 | 43.9 | 44.3 | 120 |  | Bing et al., 2013 |
|  | L3 | Jiaoganghu Lake(Freshwater) | 37.5 | 0.80 | 8.5 | 0.22 |  | 35.8 | 35.8 | 27.1 | 160.4 | 0.04 | Chen e tal., 2016 |
|  | L4 | Huanghu Lake(Freshwater) | 299.2 | 3.94 |  | 0.49 | 93.5 | 50.3 | 49.2 | 43.7 | 119.2 |  | Guo et al., 2015 |
|  | L5 | Bohu Lake(Freshwater) | 180.4 | 4.41 |  | 0.38 | 85.5 | 36.3 | 39.9 | 42.7 | 101.7 |  | Guo et al., 2015 |
|  | L6 | Wuchanghu Lake(Freshwater) | 100.5 | 3.43 |  | 0.5 | 96.9 | 49.1 | 51 | 48.3 | 133.2 |  | Guo et al., 2015 |
|  | L7 | Chengdong Lake(Freshwater) | 180.0 | 1.50 |  | 0.33 | 83.8 | 34.9 | 42.6 | 40.9 | 106.8 |  | Guo et al., 2015 |
|  | L8 | Nvshan Lake(Freshwater) | 104.6 | 1.71 |  | 0.23 | 85.8 | 41.5 | 50.7 | 34.2 | 104.4 |  | Guo et al., 2015 |
|  | L9 | Xiangjian Lake(Freshwater) | 45.0 | 0.93 |  | 0.22 | 66.6 | 26.1 | 31.9 | 28.5 | 75.7 |  | Guo et al., 2015 |
|  | L10 | Wabu Lake(Freshwater) | 160.0 | 2.42 |  | 0.35 | 87.6 | 41.6 | 45.6 | 38.2 | 115.3 |  | Guo et al., 2015 |
|  | L11 | Shengjin Lake(Freshwater) | 78.5 | 1.26 |  | 0.85 | 77.4 | 45.5 | 43.8 | 56.3 | 132.7 |  | Guo et al., 2015 |
|  | L12 | Huayuan Lake(Freshwater) | 34.0 | 1.35 |  | 0.39 | 90.6 | 38.1 | 48.2 | 49.1 | 117.4 |  | Guo et al., 2015 |
|  | L13 | Chengxi Lake(Freshwater) | 89.0 | 2.70 |  | 0.26 | 86 | 38.5 | 43.3 | 35.8 | 104 |  | Guo et al., 2015 |
|  | L14 | Tuohu Lake(Freshwater) | 40.0 | 1.20 |  | 0.3 | 70.7 | 35 | 39.5 | 31.4 | 89.9 |  | Guo et al., 2015 |
|  | L15 | Baidang Lake(Freshwater) | 39.7 | 3.06 |  | 0.44 | 85.4 | 46.7 | 45.5 | 44.1 | 119.2 |  | Guo et al., 2015 |
| Beijing | L16 | Weiminghu Lake(Freshwater) | < 1 | 1.00 |  |  | 104.0 | 12.0 |  | 26.0 | 153.6 |  | Hua et al., 2006 |
|  | L17 | Kunminghu Lake(Freshwater) | 2.2 | 1.50 |  | 1.9 | 66.5 |  |  | 28.7 |  | 0.06 | Hua et al., 2006 |
|  | L18 | Guanting Reservoir(Freshwater) | 130.0 | 10.00 |  | 0.21 | 65.5 | 31.6 | 39.5 | 33.6 | 104.0 |  | Zhang et al., 2016 |
| Guangdong | L19 | Hedi Reservoir(Freshwater) | 1440.0 | 10.10 |  | 0.71 | 51.3 | 28.4 | 22.1 | 78.4 | 345.8 | 0.18 | Wang et al., 2014b |
|  | L20 | Gaozhou Reservoir(Freshwater) | 1022.0 | 81.00 |  | 1.1 | 54.7 | 33.9 | 23.1 | 127.9 | 693.4 | 0.17 | Hu et al., 2011 |
|  | L21 | Dashuiqiao Reservoir(Freshwater) | 196.0 | 6.00 |  | 0.38 | 232.8 | 110.3 | 248.7 | 16.2 | 76.3 | 0.68 | Wang et al., 2014b |
| Guizhou | L22 | Baihuahu Lake(Freshwater) | 14.5 | - |  | 0.95 | 66.0 | 102.7 |  | 40.0 | 184.3 |  | Tian et al., 2012b; Wang et al., 2012 |
|  | L23 | Red maple lake(Freshwater) | 57.2 | 10~40 | 29.7 | 0.77 | 87.9 | 91.9 | 190.0 | 35.9 | 142.0 | 0.66 | Liu et al., 2010 |
| Heilongjiang | L24 | Wudalianchi Lake(Freshwater) | 16.4 | 6.00 |  | 0.16 | 92.07 | 32.28 | 38.35 | 24.7 | 82.73 |  | Guo et al., 2015 |
|  | L25 | Xingkai Lake(Freshwater) | 4380.0 | 4.50 |  | 0.14 | 75.95 | 19.65 | 25.35 | 21.63 | 60.35 |  | Guo et al., 2015 |
|  | L26 | Jingbo Lake(Freshwater) | 91.5 | 12.90 | 7.28 | 0.26 | 90.78 | 32.45 | 47.4 | 28.98 | 126.25 | 0.11 | Guo et al., 2015; Hua et al., 2006 |
|  | L27 | Lianhuan Lake(Freshwater) | 580.0 | 0.50 | 4.6 | 0.05 | 18.0 | 5.9 | 6.8 | 19.0 | 22.0 | 0.01 | Cheng et al., 2015 |
|  | L28 | Nanyin Reservoir(Freshwater) | 232.0 | - | 5.0 | 0.06 | 29.0 | 9.7 | 12.2 | 20.2 | 33.0 | 0.01 | Cheng et al., 2015 |
| Hebei | L29 | Bai-yang Lake(Freshwater) | 366.0 | - | 2.0 | 6.89 | 67.63 | 35.64 | 28.83 | 55.7 | 93.22 |  | Hu et al., 2011; Lu et al., 2011 |
|  | L30 | Hengshui Lake(Freshwater) | 75.0 | 8~10 | 18.58 | 0.02 | 54.87 | 26.14 |  | 20.43 | 62.37 | 0.09 | Zhang et al., 2007 |
| Henan | L31 | Suyahu Lake(Freshwater) | 239.0 | 12.00 |  | 0.41 | 75.47 | 29.95 | 36.53 | 27.35 | 112.13 |  | Zhang et al., 2013 |
|  | L32 | Wanghua Lake(Freshwater) | 5.0 | - | 9.8 | 0.1 | 50.0 | 19.0 | 23.6 | 17.9 | 51.0 | 0.01 | Cheng et al., 2015 |
| Hubei | L33 | Liangzi Lake(Freshwater) | 280.0 | 3.00 | 35.0 | 0.8 | 115.0 | 50.0 | 53.0 | 29.0 | 124.0 | 0.08 | Wang et al., 2016 |
|  | L34 | Moshui Lake(Freshwater) | 30.0 | 1.20 |  |  | 84.3 | 40.7 | 50.3 | 31.5 | 108.4 |  | Liu et al., 2008 |
|  | L35 | Honghu Lake(Freshwater) | 402.0 | 1.34 | 11.4 | 0.3 | 104.0 | 44.4 | 49.6 | 33.1 | 110.0 | 0.1 | Cheng et al., 2015 |
|  | L36 | Donghu Lake(Freshwater) | 33.0 | 2.50 | 16.03 | 0.57 | 119.22 | 75.56 | 40.44 | 57.78 | 296.78 | 0.32 | Yang et al., 2009 |
| Hunan | L37 | Dongting Lake(Freshwater) | 2820.0 | 6.39 | 29.71 | 4.65 | 88.29 | 47.48 |  | 60.99 | 185.25 | 0.16 | Li et al., 2013 |
|  | L38 | Datong Lake(Freshwater) | 114.2 | 2.89 | 34.53 | 2.38 |  |  |  | 61.95 |  | 0.28 | Zhu et al., 2008 |
|  | L39 | East Dongting Lake(Freshwater) | 1328.0 | 4~22 | 21.62 | 15.2 | 100.97 | 63.38 |  | 35.78 | 113.73 | 0.11 | Wan et al., 2011 |
| Inner Mongolia | L40 | Dalinouer Lake(Saltwater) | 238.0 | 6.80 | 11.49 | 0.17 | 58.90 | 23.66 |  | 20.26 | 67.79 | 0.03 | Zhao, 2013a |
|  | L41 | Hulun Lake(Freshwater) | 2339.0 | 5.92 |  | 0.09 | 31.37 | 16.17 | 15.61 | 20.87 | 48.43 |  | Cheng et al., 2015; Guo et al., 2015 |
|  | L42 | Ulansuhai Lake(Freshwater) | 293.0 | 0.90 | 10.55 | 0.16 | 58.54 | 27.13 | 29.79 | 20.66 | 78.38 | 0.09 | Zhao et al., 2013b; Guan et al., 2016 |
| Jiangsu | L43 | Taihu Lake(Freshwater) | 2338.1 | 1.89 | 5.94 | 0.82 | 41.5 | 27.82 | 28.75 | 41.17 | 65.46 |  | Jiang et al., 2012 |
|  | L44 | Hongze Lake(Freshwater) | 1597.0 | 1.77 | 23.67 | 3.24 | 57.59 | 34.99 | 35.17 | 18.82 | 72.44 | 0.07 | Yu et al., 2011 |
|  | L45 | Gaoyou Lake(Freshwater) | 674.0 | 3.5~4.5 | 26.86 | 0.27 | 76.09 | 39.72 | 34.0 | 25.27 | 114.1 | 0.04 | Cheng et al., 2015 |
|  | L46 | Gehu Lake(Freshwater) | 164.0 | 1.47 | 24.43 | 2.34 | 65.01 | 45.84 | 20.86 | 17.78 | 101.59 | 0.41 | Xiong et al., 2016 |
|  | L47 | Gucheng Lake(Freshwater) | 24.5 | 1.56 | 22.65 | 0.67 | 60.22 | 37.93 | 29.15 | 31.93 | 130.97 |  | Chen et al., 2013a |
|  | L48 | Luoma Lake(Freshwater) | 260.0 | 3.32 | 13.4 | 0.13 | 64.78 | 28.19 | 29.77 | 24.29 | 69.64 |  | Chen et al., 2013; Chen et al., 2013a |
|  | L49 | Baima Lake(Freshwater) | 108.0 | 0.97 | 34.90 | 0.45 | 48.67 | 25.62 | 25.10 | 29.60 | 77.30 |  | Chen et al., 2013a |
|  | L50 | Shaobo Lake(Freshwater) | 77.0 | - | 11 |  | 62 | 26 | 30 | 27 | 82 | 0.038 | Liu and Li, 2011 |
|  | L51 | Yangcheng Lake(Freshwater) | 120.0 | 1.7~3.0 |  |  | 101 | 38 | 59.1 | 42 | 138 |  | Wu et al., 2012 |
|  | L52 | Sanjiu Lake(Freshwater) | 23.2 | - | 19.89 |  | 56.93 | 60.19 | 40.63 | 21.58 | 200.78 |  | Li et al., 2016 |
|  | L53 | Chenghu Lake(Freshwater) | 45.0 | 1.83 | 10.7 | 0.41 | 97.5 | 49.2 | 52.7 | 34.2 | 119 |  | Jiang et al., 2015 |
|  | L54 | Kuncheng Lake(Freshwater) | 18.4 | 2.00 | 20.35 |  | 69.81 | 25.99 | 14.61 | 3.03 | 69.79 |  | Li et al., 2016 |
|  | L55 | Changdang Lake(Freshwater) | 89.0 | 1.15 | 9.9 | 2.98 | 80.4 | 43.2 | 42.2 | 33.9 | 121 |  | Jiang et al., 2015 |
|  | L56 | Xuanwu Lake(Freshwater) | 3.7 | < 1 | 13.1 | 0.46 | 68.85 | 41.51 | 31.85 | 35.53 | 145.29 |  | Chen et al., 2013 |
|  | L57 | Shijiu Lake(Freshwater) | 196.0 | 1.7~2.4 | 19.17 | 0.68 | 52.59 | 35.16 | 31.26 | 33.26 | 154.42 |  | Chen et al., 2013 |
| Jiangxi | L58 | Poyang Lake(Freshwater) | 4125.0 | 8.40 | 12.1 | 0.24 | 63.0 | 27.7 | 26.1 | 42.3 | 100.0 | 0.08 | Yuan et al., 2011; Cheng et al., 2015 |
| Jilin | L59 | Songhua Lake(Freshwater) | 550.0 | 30-40 |  | 0.82 | 85.6 | 137.3 | 101.2 | 34.3 | 224.1 | 2.1 | Hua et al., 2006 |
|  | L60 | Chagan Lake(Freshwater) | 345.0 | 4.00 | 6.6 | 0.08 | 38.0 | 14.5 | 16.8 | 20.4 | 40.0 | 0.02 | Cheng et al., 2015 |
|  | L61 | Changbai Lake(Freshwater) | 5.4 | 5~10 |  | 2.0 | 35.7 | 29.3 | 20.0 | 50.0 | 196.0 |  | Hua et al., 2006 |
|  | L62 | Nanhu Lake(Freshwater) | 0.9 | < 1 |  | 0.59 |  | 27.8 |  | 15.8 | 239.7 | 0.08 | Hua et al., 2006 |
| Liaoning | L63 | Dahuofang Reservoir(Freshwater) | 53.3 | 12.00 |  | 2.38 | 97.44 | 77.05 | 54.5 | 44.47 | 200.62 | 1.95 | Luo et al., 2011; Guo et al., 2015 |
| Ningxia | L64 | Sand Lake(Freshwater) | 22.0 | 1~5 | 13.73 | 0.16 | 58.6 | 21.6 | 36.7 | 21.38 | 60.46 | 0.03 | Zhu et al., 2013a |
|  | L65 | Hequan Lake(Freshwater) | 2.0 | 1~5 | 7.91 | 0.3 | 63.76 | 26.44 | 31.72 | 25.48 | 77.45 | 0.05 | Zhu et al., 2013a |
|  | L66 | Yuehai Lake(Freshwater) | 30.0 | 1~5 | 11.2 | 0.18 | 63.27 | 24.41 | 29.04 | 22.65 | 68.89 | 0.03 | Zhu et al., 2013a |
|  | L67 | Tianhu Lake(Freshwater) | - | 1~5 | 7.5 | 0.17 | 61.56 | 20.87 | 27.77 | 19.88 | 60.35 | 0.01 | Zhu et al., 2013a |
| Qinghai | L68 | Qinghai Lake(Saltwater) | 4400.0 | 21.70 | 10.8 | 0.18 | 64.8 | 23.7 | 18.09 | 23.61 | 86.35 |  | Zhu et al., 2013b |
| Shanghai | L69 | Dianshan Lake(Freshwater) | 62.0 | 2.63 | 8.89 |  | 50.89 | 16.14 | 20.46 | 42.91 | 126.31 |  | Xu et al., 2011 |
|  | L70 | Dishui Lake(Freshwater) | 5.6 | 3.70 | 6.97 | 0.17 | 77.12 | 29.35 |  | 19.11 | 86.28 | 0.17 | Liu et al., 2016 |
| Shaanxi | L71 | Hongjiannao Lake(Freshwater) | 32.9 | 8.20 | 10.16 | 0.17 | 55.85 | 19.27 |  | 19.72 | 48.98 | 0.02 | Yu et al., 2016 |
| Sichuan | L72 | Qionghai Lake(Freshwater) | 31.0 | 10.32 | 8.71 | 0.7 | 88.06 | 32.04 | 45.32 | 45.51 | 141.8 | 0.05 | Song et al., 2014 |
|  | L73 | Mahu Lake(Freshwater) | 7.5 | 70.00 |  | 0.81 | 172.86 | 135.73 | 62.44 | 40.01 | 156.71 |  | Guo et al., 2015 |
|  | L74 | Yihai Lake(Freshwater) | 1.0 | 9.80 |  | 1.08 | 41.63 | 18.43 | 19.6 | 58.78 | 109.7 |  | Guo et al., 2015 |
|  | L75 | Daqiao Reservoir(Freshwater) | 23.5 | 30.00 |  | 0.61 | 166.35 | 86.65 | 95.88 | 58.27 | 159 |  | Guo et al., 2015 |
| Shandong | L76 | Dongping Lake(Freshwater) | 148.0 | 1.59 | 25.3 | 0.29 | 89.3 | 52 |  | 35.5 | 100.5 | 0.06 | Wang et al., 2015 |
|  | L77 | Nansi Lake(Freshwater) | 1266.0 | 1.46 | 17.7 | 0.23 | 85.5 | 37 | 39.3 | 28.7 | 87.5 | 0.05 | Wang et al., 2014a |
| Tibet | L78 | Wuran Co(Freshwater) | 22.0 | < 6 | 36.5 | 0.21 | 55.9 | 43.9 | 31.5 | 76.7 | 144 |  | Guo et al., 2016 |
|  | L79 | Lang Co(Saltwater) | 12.1 | - | 28 | 0.26 | 110 | 74.1 | 69.1 | 27.2 | 123 |  | Guo et al., 2016 |
|  | L80 | Gongzhu Co(Saltwater) | 66.2 | - | 216 | 0.04 | 262 | 41.4 | 44.5 | 13.2 | 53.9 |  | Guo et al., 2016 |
|  | L81 | Bangong Co(Saltwater) | 604.0 | 5.00 | 14.5 | 0.06 | 164 | 32.9 | 38.5 | 20.4 | 109 |  | Guo et al., 2016 |
|  | L82 | Songmuxi Co(Freshwater) | 24.6 | - | 34.8 | 0.26 | 56.5 | 28.9 | 34.9 | 53.7 | 156 |  | Guo et al., 2016 |
|  | L83 | Longmu Co(Salt) | 97.0 | - | 11.1 | 0.1 | 11.9 | 9.17 | 11.2 | 22.8 | 46.8 |  | Guo et al., 2016 |
|  | L84 | Aiyong Co(Salt) | 22.4 | - | 65.8 | 0.02 | 35.7 | 12.7 | 35.6 | 12.2 | 53.2 |  | Guo et al., 2016 |
|  | L85 | Lubu Co(-) | 12.4 | - | 12.75 | 0.32 | 78.9 | 14.75 | 21.4 | 21.95 | 47.25 |  | Guo et al., 2016 |
|  | L86 | Kunzhong Co(-) | 15.0 | - | 16.2 | 0.25 | 102.3 | 32.5 | 45.6 | 17.25 | 64.7 |  | Guo et al., 2016 |
|  | L87 | Rebang Co(Salt) | 31.6 | - | 53.3 | 0.83 | 72.7 | 21.6 | 95.2 | 40.7 | 64.4 |  | Guo et al., 2016 |
|  | L88 | Aweng Co(Salt) | 58.6 | - | 29.1 | 0.27 | 40.8 | 17.5 | 58.7 | 20.3 | 49.0 |  | Guo et al., 2016 |
|  | L89 | Chagcam Caka(Salt) | 128.3 | 0.15~1.2 | 153.8 | 0.24 | 29.53 | 11.71 | 12.07 | 16.9 | 42.83 |  | Guo et al., 2016 |
|  | L90 | Bieruoze Co(Salt) | 33.2 | - | 463 | 0.65 | 38.7 | 12.9 | 20.9 | 16.0 | 47.1 |  | Guo et al., 2016 |
|  | L91 | Darebu Co(-) | 21.0 | - | 31.8 | 0.03 | 100.0 | 14.3 | 11.0 | 13.1 | 31.2 |  | Guo et al., 2016 |
|  | L92 | Dong Co(Saltwater) | 123.5 | - | 53.1 | 0.26 | 26.5 | 9.79 | 22.8 | 26.2 | 42.6 |  | Guo et al., 2016 |
|  | L93 | Daze Co(Saltwater) | 244.7 | < 38 | 27.1 | 0.6 | 63.8 | 18.95 | 33.2 | 18.5 | 65.8 |  | Guo et al., 2016 |
|  | L94 | Pumoyong Co(Freshwater) | 290.0 | - |  | 0.13 | 73.72 | 42.3 | 32.08 | 47.67 | 142.1 |  | Guo et al., 2016 |
|  | L95 | Yangzhuoyong Co(Saltwater) | 638.0 | 30.00 | 48.26 | 0.08 | 67.39 | 31.48 | 35.28 | 21.72 | 62.53 |  | Guo et al., 2015 |
|  | L96 | Basong Co(Freshwater) | 25.5 | 25.00 |  | 0.61 | 95.88 | 52.36 | 51.8 | 81.7 | 174.4 |  | Guo et al., 2015 |
|  | L97 | Namu Co(Saltwater) | 1961.5 | 33.00 |  | 0.17 | 38.4 | 18.7 | 23.0 | 25.1 | 57.6 |  | Guo et al., 2015 |
| Xinjiang | L98 | Jili Lake(Freshwater) | 174.0 | 9.90 |  | 0.08 | 36.73 | 14.07 | 16.35 | 18.1 | 37.53 |  | Guo et al., 2015 |
|  | L99 | Wulungu Lake(Freshwater) | 753.0 | 8.00 |  | 0.14 | 60.63 | 29.75 | 30.53 | 19.45 | 66.5 |  | Guo et al., 2015 |
|  | L100 | Bositeng Lake(Freshwater) | 992.2 | 8.08 |  | 0.14 | 46.4 | 21.64 | 27.36 | 16.71 | 51.09 |  | Guo et al., 2015 |
|  | L101 | Aibi Lake(Saltwater) | 550.0 | 1~2.5 | 15.57 | 0.17 | 51.49 | 39.89 | 28.27 | 39.57 | 114.59 | 0.03 | Zhang et al., 2015 |
| Yunnan | L102 | Dianchi Lake(Freshwater) | 297.9 | 4.40 | 31.86 | 1.87 | 105.73 | 83.63 | 54.92 | 65.68 | 205.64 | 0.24 | Li et al., 2012 |
|  | L103 | Erhai Lake(Freshwater) | 256.5 | 10.00 | 26.9 | 1.1 | 103.8 | 63.1 | 52.2 | 47.4 | 109.0 | 0.17 | Lin et al., 2016 |
|  | L104 | Qilu Lake(Freshwater) | 36.9 | 4.03 |  | 1.0 | 118.6 | 76.75 | 55.53 | 41.43 | 128.77 |  | Guo et al., 2015 |
|  | L105 | Lugu Lake(Freshwater) | 48.5 | 40.30 |  | 0.61 | 161.6 | 128.66 | 102.78 | 29.42 | 148.0 |  | Guo et al., 2015 |
|  | L106 | Chenghai Lake(Freshwater) | 77.8 | 25.70 |  | 0.66 | 98.32 | 59.37 | 54.48 | 31.9 | 119.0 |  | Guo et al., 2015 |
|  | L107 | Xingyun Lake(Freshwater) | 34.7 | 7.00 | 9.2 | 0.49 | 109.0 | 59.6 | 39.8 | 34.8 | 99.0 | 0.05 | Cheng et al., 2015 |
|  | L108 | Fuxian Lake(Freshwater) | 216.6 | 95.20 | 11.5 | 0.6 | 86.0 | 68.4 | 41.6 | 78.2 | 121.0 | 0.11 | Cheng et al., 2015 |
| Zhejiang | L109 | West Lake(Freshwater) | 6.4 | 2.28 | 17.75 | 0.43 | 68.5 | 20.6 |  |  | 88.6 | 1.5 | Hua et al., 2006 |
|  | L110 | Dongqian Lake(Freshwater) | 19.9 | 2.00 | 8.14 | 0.99 | 51.2 | 28.4 |  | 43.1 |  | 0.03 | Cheng et al., 2007 |

**Table S2 Materials and methods of heavy metal contamination in sediment from lakes in China.**

| Lakes (Sampling depth / cm) | Digestion | Analysis method | Reference |
| --- | --- | --- | --- |
| Longganghu Lake(0-20); Luoma Lake(0-20); Xuanwu Lake(0-20); Shijiu Lake(0-20); Qinghai Lake(0-20); Dianchi Lake(0-15)；Jiaoganghu Lake(0-10); Shaobo Lake(0-20); Yangcheng Lake(0-10) | HF+HCLO_4_+HNO_3_ | ICP-AES | Bing et al., 2013; Chen et al., 2013; Zhu et al., 2013a; Li et al., 2012; Chen et al., 2016; Liu and Li, 2011; Wu et al., 2012 |
| Chaohu Lake(0-10) | HF+HNO_3_+HCL | ICP-OES | Yin et al., 2011; Hua et al., 2006 |
| Huanghu Lake(0-20); Bohu Lake(0-20); Wuchanghu Lake(0-20); Chengdong Lake(0-20); Nvshan Lake(0-20); Xiangjian Lake(0-20); Wabu Lake(0-20); Shengjin Lake(0-20); Huayuan Lake(0-20); Chengxi Lake(0-20); Tuohu Lake(0-20); Baidang Lake(0-20); Wudalianchi Lake(0-20); Xingkai Lake(0-20); Jingbo Lake(0-20);  Mahu Lake(0-20); Yihai Lake(0-20); Daqiao Reservoir(0-20); Yangzhuoyong Co(0-20); Basong Co(0-20); Namu Co(0-20); Jili Lake(0-20); Wulungu Lake(0-20); Bositeng Lake(0-20); Qilu Lake(0-20);  Lugu Lake(0-20); Chenghai Lake(0-20) | HF+HCLO_4_+HNO_3_ | ICP-AES+ ICP-OES | Guo et al., 2015; Guo et al., 2016 |
| Weiminghu Lake(0-10); Kunminghu Lake(0-10); Bai-yang Lake(0-20); Hengshui Lake(0-5); Suyahu Lake(0-20); Ulansuhai Lake(0-5); Hongze Lake(0-20); Songhua Lake(0-20); Changbai Lake(0-20); Nanhu Lake(0-20); Qionghai Lake(0-20); West Lake(0-20) | HF+HCLO_4_+HNO_3_ | AAS | Hua et al., 2006; Hu et al., 2011; Lu et al., 2011;  Zhang et al., 2007; Zhang et al., 2013; Zhao et al., 2013b; Zhu et al., 2013b; Yu et al., 2011; Song et al., 2014 |
| Guanting Reservoir(0-20) | HF+HCLO_4_+HNO_3_ | ICP-OES+ ICP-MS | Zhang et al., 2016 |
| Hedi Reservoir(0-20); DashuiqiaoReservoir (0-20); Liangzi Lake(0-10); East Dongting Lake(0-10);  Taihu Lake(5); Dahuofang Reservoir(0-5); Wuran Co(0-20); Lang Co(0-20); Gongzhu Co(0-20); Bangong Co(0-20); Songmuxi Co(0-20); Longmu Co(0-20); Aiyong Co(0-20); Lubu Co(0-20); Kunzhong Co(0-20); Rebang Co(0-20); Aweng Co(0-20); Chagcam Caka(0-20); Bieruoze Co(0-20); Darebu Co(0-20); Dong Co(0-20); Daze Co(0-20); Pumoyong Co(0-20); Yangzhuoyong Co(0-20); Basong Co(0-20); Namu Co(0-20) | HF+HNO_3_ | ICP-MS | Wang et al., 2014b; Wang et al., 2016; Wan et al., 2011; Jiang et al., 2012; Luo et al., 2011; Guo et al., 2016 |
| Gaozhou Reservoir(0-20) | HF+HCLO_4_+HNO_3_ | AAS+AFS | Hu et al., 2011 |
| Baihuahu Lake(0-10); Dongqian Lake(0-20) | HF+HCLO_4_+HNO_3_ | AAS+AFS | Tian et al., 2012b; Wang et al., 2012; Cheng et al., 2007 |
| Lianhuan Lake(0-20); Nanyin Reservoir(0-20); Wanghua Lake(0-20); Honghu Lake(0-20); Xingyun Lake(0-20); Fuxian Lake(0-20) Hulun Lake(0-20); Gaoyou Lake(0-20);Chagan Lake(0-20); Nansi Lake(0-5) | HF+HCLO_4_+HNO_3_+aqua regia | AFS+XFR | Cheng et al., 2015; Wang et al., 2014a |
| Donghu Lake(0-20) | HF+HCLO_4_+HNO_3_+H_2_SO_4_ | ICP-MS+CVAFS | Yang et al., 2009; |
| Dongting Lake(0-20); Gehu Lake(0-15); Sand lake(0-20); Hequan Lake(0-20); Yuehai Lake(0-20); Tianhu Lake(0-20); Hongjiannao Lake(0-10) | HF+HCLO_4_+HNO_3_ | ICP-MS+AFS | Li et al., 2013; Xiong et al., 2016; Zhu et al., 2013a; Yu et al., 2016; |
| Datong Lake(0-10); Dishui Lake(0-10) | HF+HCLO_4_+HNO_3_+H_2_SO_4_+HCL | AAS+AFS | Zhu et al., 2008; Liu et al., 2016; |
| Gucheng Lake(0-20); Baima Lake(0-20) | HF+HCLO_4_+HNO_3_+HCL | OPTIMA(ICP) | Chen et al., 2013a |
| Poyang Lake(0-25); Dianshan Lake(0-15) | HF+HCLO_4_+HNO_3_ | AAS+XRF | Yuan et al., 2011; Xu et al., 2011 |
| Dongping Lake(0-20); Dalinouer Lake(0-20) | HF+HCLO_4_+HNO_3_ | ICP-AES+AFS | Wang et al., 2015; Zhao, 2013a |
| Erhai Lake(0-2); Chenghu Lake(0-5); Changdang Lake(0-5) | HF+HCLO_4_+HNO_3_+HCL | ICP-AES+ICP-MS | Lin et al., 2016; Jiang et al., 2015 |
| Moshui Lake(0-20) | HF+HCLO_4_ | ICP-OES | Liu et al., 2008 |
| Sanjiu Lake(0-20); Kuncheng Lake(0-20) | HF+aqua regia | ICP-MS | Li et al., 2016 |

**References for Table S1 and S2**

Bing, H. J., Wu, Y. H., Liu, E. F., Yang, X. D. (2013). Assessment of heavy metal enrichment and its human impact in lacustrine sediments from four lakes in the mid-low reaches of the Yangtze River, China. Journal of Environmental Sciences, 25(7): 1300-1309.

Chen, Q. K., Liu, T., Hu, Z. X., Shi, F., Hu, X., Yang, L. Y. (2013a). Distribution and ecological risk assessment of heavy metal in surface sediments from lake of west Jiangsu province. Journal of Agro-Environment Science, 32(5): 1044-1050.(in Chinese)

Chen, P. X., Wang, B., Sun, Q. Y. (2016). Potential ecological risk assessment of heavy metals in surface sediments of Jiaogang Lake and its Inflow rivers. Wetland Science, 14(1): 37-43.(in Chinese)

Cheng, N. N., Li, W., Ran, G. X., Du, X. H., Cao, H. (2007). Characteristics and risk evaluation of pollutants in sediments of Lake Dongqian, Zhejiang Province. Journal of Lake Sciences, 19(1): 58-62. (in Chinese)

Cheng, H. G., Li, M., Zhao, C. D., Yang, K., Li, K., Peng, M., Yang, Z. F., Liu, F., Liu, Y. H., Bai, R. J., ... , Yin, G. S. (2015). Concentrations of toxic metals and ecological risk assessment for sediments of major freshwater lakes in China. Journal of Geochemical Exploration, 157: 15-26.

Guan, Q. Y., Wang, L., Pan, B. T., Guan, W. Q., Sun, X. Z., Cai, A. (2016). Distribution features and controls of heavy metals in surface sediments from the riverbed of the Ningxia-Inner Mongolian reaches, Yellow River, China. Chemosphere, 144: 29-42.

Guo, B. X., Liu, Y. Q., Zhang, F., Hou, J. Z., Zhang, H. B. (2016). Characteristics and risk assessment of heavy metals in core sediments from lakes in Tibet. Environmental Science, 37(2): 490-498.(in Chinese)

Guo, W., Huo, S. L., Xi, B. D., Zhang, J. T., Wu, F. C. (2015). Heavy metal contamination in sediments from typical lakes in the five geographic regions of China: distribution, bioavailability, and risk. Ecological Engineering, 81: 243-255.

Hua, L. P., Hua, L., Gao, J., Zhang, Z. X., Yin, X. X., Zhu, F. Y., Wang, X. D. (2006). Heavy metal pollution of sediments of lakes in China. Soils, 38(4): 366-373.(in Chinese)

Hu, G. C., Xu, M. Q., Xu, Z. C., Dai, J. Y., Cao, H., Peng, X. W., Qi, J. Y. (2011). Pollution characteristic and potential risk assessment of heavy metals in surface sediment from Fuhe River and Baiyangdian Lake, north China. Journal of Agro-Environment Science, 30(1): 146-153.(in Chinese)

Jiang, X., Wang, W. W., Wang, S. H., Zhang, B., Hu, J. C. (2012). Intial identification of heavy metals contamination in Taihu Lake, a eutrophic lake in China. Journal of Environmental science, 24: 1539-1548.

Jiang, Y,, Liu, X., Gao, J. F., Cai, Y., J. (2015). Pollution characteristics and potential ecological risk assessment of heavy metals in sediments of shallow lakes in Jiangsu province, China. Resources and Environment in Yangtze Basin, 24(7): 1157-1162.(in Chinese)

Li, F., Huang, J. H., Zeng, G. M., Yuan, X. Z., Li, X. D., Liang, J., Wang, X. Y., Tang, X. J., Bai, B. (2013). Spatial risk assessment and sources identification of heavy metals in surface sediments from the Dongting Lake, Middle China. Journal of Geochemical Exploration, 132: 75-83.

Lin, Q. F., Liu, E., Zhang, E. L., Li, K., Shen, J. (2016). Spatial distribution, contamination and ecological risk assessment of heavy metals in surface sediments of Erhai Lake, a large eutrophic plateau lake in southwest China. Catena, 145: 193-203.

Liu, C., Bi, C. J., Tao, Z. K., Chen, Z. L. (2016). Seasonal variation of heavy metals in surface sediment of the Dishui Lake and its potential ecological risk. Urban Environment & Urban Ecology, 29(2): 38-42.(in Chinese)

Liu, F., Hu, J. W., Qin, F. X., Wu, D., Li, C. X., Huang, X. F., Jiang, G. H. (2010). An assessment of heavy metal contamination sources in sediments from Hongfeng Lake. Acta Scientiae Circumstantiae, 30(9): 1871-1879.(in Chinese)

Liu, H. L., Li, L. Q., Yin, C. Q., Shan, B. Q. (2008). Fraction distribution and risk assessment of heavy metals in sediments of Moshui Lake. Journal of Environmental Sciences, 20(4): 390-397.

Liu, H., Li, W. (2011). Dissolved trace elements and heavy metals from the shallow lakes in the middle and lower reaches of the Yangtze River region, China. Environmental Earth Sciences, 62(7): 1503-1511.

Li, Y. B., Feng, L., Liu, Z. T., Zhou, Z. L. (2012). Ecological risk assessment of sediment heavy metals in main lakes of China. Environmental Science & Technology, 35(2): 200-205.(in Chinese)

Li, Y. J., Zhang, L. Y., Wu, Y. W., Li, C. L., Yang, T. X., Tang, J. (2016). GIS spatial distribution and ecological risk assessment of heavy metals in surface sediments of shallow lakes in Jiangsu province. Environmental Science, 37(4): 1321-1329.(in Chinese)

Lu, C. X., Cheng, J. M. (2011). Speciation of Heavy Metals in the Sediments from Different Eutrophic Lakes of China. Procedia Engineering, 18: 318-323.

Luo, Y., Qin, Y. W., Zhang, L., Zheng, B. H., Jia, J. (2011). Analysis and assessment of heavy metal pollution in surface of the Dahuofang reservoir. Acta Scientiae Circumstantiae, 31(5): 987-995.(in Chinese)

Song, X. B., Shi, Z. M., Kan, Z. Z., Liao, J. X., Yang, W. H., Zhang, J. J. (2014). Source identification and hazardous risk delineation of heavy metals in surface sediments from Lake Qinghai, Sichuan province. Earth and Environment, 42(4): 532-539.(in Chinese)

Tian, L. F., Hu, J. W., Luo, G. L., Ma, J. J., Huang, X. F., Qin, F. X. (2012b). Ecological risk and stability of heavy metals in sediments from lake Baihua in Guizhou province. Acta Scientiae Circumstantiae, 32(4): 885-894.(in Chinese)

Wang, D., Meng, X., Zhang, H., Gao, Z. J., Shan, B. Q., Li, S. M. (2016). Pollution analysis and ecology risk assessment in sediments of Liangzi Lake. Acta Scientiae Circumstantiae, 36(6): 1901-1909.(in Chinese)

Wang, L. F., Yang, L. Y., Kong, L. H., Li, S., Zhu, J. R., Wang, Y. Q. (2014a). Spatial distribution, source identification and pollution assessment of metal content in the surface sediments of Nansi Lake, China. Journal of Geochemical Exploration, 140: 87-95.

Wang, M., Wang, S., Tang, Q. H., Zhang, H. J., Luo, C., Wei, G. F., Peng, L., Yang, H. W. (2014b). Characteristics of sediment Nutrients loading and heavy metals pollution in three important reservoirs from the west coast of Guangdong province, south China. Ecology and Environmental Sciences, 23(5): 834-841.(in Chinese)

Wang, Y., Hu, J. W., Xiong, K. G., Huang, X. F., Duan, S. M. (2012). Distribution of heavy metals in core sediments from Baihua Lake. Procedia Environmental Sciences, 16: 51-58.

Wang, Y. Q., Yang, L. Y., Kong, L. H., Liu, E. F., Wang, L. F., Zhu, J. R. (2015). Spatial distribution, ecological risk assessment and source identification for heavy metals in surface sediments from Dongping Lake, Shandong, East China. Catena, 125: 200-205.

Wan, Q., Li, F., Zhu, H. N., Wang, T., Liang, J., Huang, J. H., Xie, G. X. (2011). Distribution characteristics, pollution assessment and source identification of heavy metals in sediment of east Dongting lake. Research of Environmental Science, 24(12): 1378-1384.(in Chinese)

Wu, J. L., Zeng, H. A., Yu, H., Ma, L., Xu, L. S., Qin, B. Q. (2012). Water and sediment quality in lakes along the middle and lower reaches of the Yangtze River, China. Water resources management, 26(12): 3601-3618.

Xiong, C. H., Zhang, R. L., Wu, X. D., Feng, L. H., Wang, L. Q. (2016). Distribution and pollution risk assessment of nutrient and heavy metals in surface sediments from lake Gehu in southern Jiangsu province, China. Environmental Science, 3: 925-934.(in Chinese)

Xu, L. L., Ma, C. A., Tian, W., Lü, W. W., Zhao, Y. L. (2011). The distribution of heavy metals in surface sediment of lake Dianshan and its correlations with macrozoobenthos. Acta Scientiae Circumstantiae, 31(10): 2223-2232.(in Chinese)

Yang, Z. F., Wang, Y., Shen, Z. Y., Niu, J. F., Tang, Z. W. (2009). Distribution and speciation of heavy metals in sediments from the mainstream, tributaries, and lakes of the Yangtze River catchment of Wuhan, China. Journal of Hazardous Materials, 166(2): 1186-1194.

Yin, H. B., Deng, J. C., Shao S. G., Gao, F., Gao, J. F., Fan, C. X. (2011). Distribution characteristics and toxicity assessment of heavy metals in the sediments of Lake Chaohu, China. Environmental Monitoring and Assessment, 179(1): 431-442.

Yu, H., Zhang, W. B., Yu, J. P. (2011). Distribution and potential ecological risk assessment of heavy metals in surface sediments of Hongze Lake. Environmental Science, 32(2): 437-444.(in Chinese)

Yu, X. F., Liu, X. Q., Wang, Y. P. (2016). Ecological risk assessment of heavy metals in the surface sediment of Hongjiannao Lake, Shaanxi Province, China. Journal of Earth Environment, 7(2): 173-182.(in Chinese)

Yuan, G. L., Liu, C., Chen, L., Yang, Z.F. (2011). Inputting history of heavy metals into the inland lake recorded in sediment profiles: Poyang Lake in China. Journal of hazardous materials, 185(1): 336-345.

Zhang, B. Z., Wang, D., Zhang, H., Meng, X., Lei, P., Zhu, X. L., Shan, B. Q. (2016). The flux of sedimentary heavy metals and variation of ecological risks recorded by sediments from Guanting Reservoir. Acta Scientiae Circumstantiae, 36(2): 458-465.(in Chinese)

Zhang, M. Y., Cui, L. J., Sheng, L. X., Wang, Y. F. (2007). Pollution ecological risk assessment of heavy metals in the Hengshuihu wetland. Wetland Science, 5(4): 362-369.(in Chinese)

Zhang, P. Y., Zhao, Y. F., Chen, Y. Z., Qin, M. Z., Chen, L., Hu, C. H. (2013). Content of heavy metals and spatial differences in sediments of lake bottom in Suya Lake, near Zhumadian, Henan. Journal of Safety and Environment, 13(2): 125-129.(in Chinese)

Zhang, Z. Y., Jilili, A., Jiang, F. Q. (2015). Sources, pollution statue and potential ecological risk of heavy metals in surface sediments of Aibi Lake, northwest China. Environmental Science, 36(2): 490-496.(in Chinese)

Zhao, S. Z. (2013a). A study of nutrient elements and heavy matal pollitants and their enviromental effects of the water and bed sediments of the Wuliangsu lake in Inner Megolia[D]. Groundwater Science and Engineering, China University of GeoSciences: 35.(in Chinese)

Zhao, S. N., Li, C. Y., Shi, X. H., Zhang, H. M., Wang, S. (2013b). Bioavailability and environment pollution evaluation of sediments heavy metals in Wuliangsuhai lake. Ecology and Environmental Sciences, 22(3): 481-489.(in Chinese)

Zhu, W., Li, Z. G., Li, J., Li, C. X., Li, Z. J., Yang, Y. M. (2013a). Pollution characteristics and potential ecological risk assessment of heavy metals in the lake wetlands in the Yellow River Valleys of Ningxia. Chinese Agriculture Science Bulletin, 29(35): 281-288.(in Chinese)

Zhu, Y. C., Chen, X. M., Fu, X. Y. (2013b). Enrichment characteristics and sources of heavy metals in surface sediments of Qinghai Lake, Northwest China. Chinese Journal of Ecology, 32(7): 1862-1869.(in Chinese)

Zhu, Y. L., Jiang, J. H., Huang, Q., Sun, Z. D., Wang, H. J., Zhou, Y. K. (2008). Contents, Distribution and Correlation of Cd, Pb, Hg, As in Water, Sediment and Organsims from East Dongting Lake and Datong Lake. Journal of Agro-Environment Science, 27(4): 1377-1384.(in Chinese)

**Table S3 The concentrations (mg/kg) for the eight heavy metals from the researched lake sediments in China.(BVSC: Background values for soils in China; GIEQSSC: Grade I environmental quality standard for soils in China; GIIEQSSC: Grade II environmental quality standard for soils in China)**

|  | As | Cd | Cr | Cu | Ni | Pb | Zn | Hg |
| --- | --- | --- | --- | --- | --- | --- | --- | --- |
| Min | 2 | 0.02 | 11.9 | 5.9 | 6.8 | 3.03 | 22 | 0.01 |
| Max | 463 | 15.2 | 262 | 137.3 | 248.7 | 127.9 | 693.4 | 2.1 |
| Median | 16.12 | 0.34 | 69.81 | 32.50 | 35.60 | 31.45 | 104.40 | 0.08 |
| Mean | 30.82 | 0.78 | 77.38 | 39.02 | 41.61 | 34.50 | 113.34 | 0.24 |
| BVSC | 11.2 | 0.097 | 61 | 22.6 | 26.9 | 26 | 74.2 | 0.065 |
| GIEQSSC | 15 | 0.2 | 90 | 35 | 40 | 35 | 100 | 0.15 |
| GIIEQSSC | 30 | 0.6 | 200 | 100 | 50 | 300 | 250 | 0.5 |

**Table S4 Background concentration values (mg/kg) of heavy metals in the soils of certain provinces in China**

| Province | As | Cd | Cr | Cu | Ni | Pb | Zn | Hg |
| --- | --- | --- | --- | --- | --- | --- | --- | --- |
| Anhui | 9.0 | 0.097 | 66.5 | 20.4 | 29.8 | 26.6 | 62.0 | 0.033 |
| Beijing | 9.7 | 0.074 | 68.1 | 23.6 | 29.0 | 25.4 | 102.6 | 0.069 |
| Guangdong | 8.9 | 0.056 | 50.5 | 17.0 | 14.4 | 36.0 | 47.3 | 0.078 |
| Guizhou | 20.0 | 0.659 | 95.9 | 32.0 | 39.1 | 35.2 | 99.5 | 0.11 |
| Heilongjiang | 7.3 | 0.086 | 58.6 | 20.0 | 22.8 | 24.2 | 70.7 | 0.037 |
| Hebei | 13.6 | 0.094 | 68.3 | 21.8 | 30.8 | 21.5 | 78.4 | 0.036 |
| Henan | 11.4 | 0.074 | 63.8 | 19.7 | 26.7 | 19.6 | 60.1 | 0.034 |
| Hubei | 12.3 | 0.172 | 86.0 | 30.7 | 37.3 | 26.7 | 83.6 | 0.080 |
| Hunan | 15.7 | 0.126 | 71.4 | 27.3 | 31.9 | 29.7 | 94.4 | 0.116 |
| Inner Mongolia | 7.5 | 0.053 | 41.4 | 14.4 | 19.5 | 17.2 | 59.1 | 0.040 |
| Jiangsu | 10.0 | 0.126 | 77.8 | 22.3 | 26.7 | 26.2 | 62.6 | 0.289 |
| Jiangxi | 14.9 | 0.108 | 45.9 | 20.3 | 18.9 | 32.3 | 69.4 | 0.084 |
| Jilin | 8.0 | 0.099 | 46.7 | 17.1 | 21.4 | 28.8 | 80.4 | 0.037 |
| Liaoning | 8.8 | 0.108 | 57.9 | 19.8 | 25.6 | 21.4 | 63.5 | 0.037 |
| Ningxia | 11.9 | 0.112 | 60.0 | 22.1 | 36.5 | 20.6 | 58.8 | 0.021 |
| Qinghai | 14.0 | 0.137 | 70.1 | 22.2 | 29.6 | 20.9 | 80.3 | 0.020 |
| Shanghai | 9.1 | 0.138 | 70.2 | 27.2 | 29.9 | 25.0 | 81.3 | 0.095 |
| Shaanxi | 11.1 | 0.094 | 62.5 | 21.4 | 28.8 | 21.4 | 69.4 | 0.030 |
| Sichuan | 10.4 | 0.079 | 79.0 | 31.3 | 32.6 | 30.9 | 86.5 | 0.061 |
| Shandong | 9.3 | 0.084 | 66.0 | 24.0 | 25.8 | 25.8 | 63.5 | 0.019 |
| Tibet | 19.7 | 0.081 | 76.6 | 21.9 | 32.1 | 29.1 | 74.0 | 0.024 |
| Xinjiang | 11.2 | 0.120 | 49.3 | 26.7 | 26.6 | 19.4 | 68.8 | 0.017 |
| Yunnan | 18.4 | 0.218 | 65.2 | 46.3 | 42.5 | 40.6 | 89.7 | 0.058 |
| Zhejiang | 9.2 | 0.070 | 52.9 | 17.6 | 24.6 | 23.7 | 70.6 | 0.086 |

**Table S5 I_geo_ values of heavy metals in sediments from different lakes in China.**

| Lake | As | Cd | Cr | Cu | Ni | Pb | Zn | Hg |
| --- | --- | --- | --- | --- | --- | --- | --- | --- |
| L1 | 0.58 | 1.56 | -0.81 | -0.22 | -0.43 | 0.32 | 0.72 | 2.28 |
| L2 |  | 1.35 | -0.22 | 0.73 | -0.03 | 0.15 | 0.37 |  |
| L3 | -0.67 | 0.60 |  | 0.23 | -0.32 | -0.56 | 0.79 | -0.31 |
| L4 |  | 1.75 | -0.09 | 0.72 | 0.14 | 0.13 | 0.36 |  |
| L5 |  | 1.38 | -0.22 | 0.25 | -0.16 | 0.10 | 0.13 |  |
| L6 |  | 1.78 | -0.04 | 0.68 | 0.19 | 0.28 | 0.52 |  |
| L7 |  | 1.18 | -0.25 | 0.19 | -0.07 | 0.04 | 0.20 |  |
| L8 |  | 0.66 | -0.22 | 0.44 | 0.18 | -0.22 | 0.17 |  |
| L9 |  | 0.60 | -0.58 | -0.23 | -0.49 | -0.49 | -0.30 |  |
| L10 |  | 1.27 | -0.19 | 0.44 | 0.03 | -0.06 | 0.31 |  |
| L11 |  | 2.55 | -0.37 | 0.57 | -0.03 | 0.50 | 0.51 |  |
| L12 |  | 1.42 | -0.14 | 0.32 | 0.11 | 0.30 | 0.34 |  |
| L13 |  | 0.84 | -0.21 | 0.33 | -0.05 | -0.16 | 0.16 |  |
| L14 |  | 1.04 | -0.50 | 0.19 | -0.18 | -0.35 | -0.05 |  |
| L15 |  | 1.60 | -0.22 | 0.61 | 0.03 | 0.14 | 0.36 |  |
| L16 |  |  | 0.03 | -1.56 |  | -0.55 | 0.00 |  |
| L17 |  | 4.10 | -0.62 |  |  | -0.41 |  | -0.79 |
| L18 |  | 0.92 | -0.64 | -0.16 | -0.14 | -0.18 | -0.57 |  |
| L19 |  | 3.08 | -0.56 | 0.16 | 0.03 | 0.54 | 2.29 | 0.62 |
| L20 |  | 3.71 | -0.47 | 0.41 | 0.10 | 1.24 | 3.29 | 0.54 |
| L21 |  | 2.18 | 1.62 | 2.11 | 3.53 | -1.74 | 0.10 | 2.54 |
| L22 |  | -0.06 | -1.12 | 1.10 |  | -0.40 | 0.30 |  |
| L23 | -0.01 | -0.36 | -0.71 | 0.94 | 1.70 | -0.56 | -0.07 | 2.00 |
| L24 |  | 0.31 | 0.07 | 0.11 | 0.17 | -0.56 | -0.36 |  |
| L25 |  | 0.12 | -0.21 | -0.61 | -0.43 | -0.75 | -0.81 |  |
| L26 | -0.59 | 1.01 | 0.05 | 0.11 | 0.47 | -0.32 | 0.25 | 0.99 |
| L27 | -1.25 | -1.37 | -2.29 | -2.35 | -2.33 | -0.93 | -2.27 | -2.47 |
| L28 | -1.13 | -1.10 | -1.60 | -1.63 | -1.49 | -0.85 | -1.68 | -2.47 |
| L29 | -3.35 | 5.61 | -0.60 | 0.12 | -0.68 | 0.79 | -0.34 |  |
| L30 | -0.13 | -2.82 | -0.90 | -0.32 |  | -0.66 | -0.91 | 0.74 |
| L31 |  | 1.89 | -0.34 | 0.02 | -0.13 | -0.10 | 0.31 |  |
| L32 | -0.80 | -0.15 | -0.94 | -0.64 | -0.76 | -0.72 | -0.82 | -2.35 |
| L33 | 0.92 | 1.63 | -0.17 | 0.12 | -0.08 | -0.47 | -0.02 | -0.58 |
| L34 |  |  | -0.61 | -0.18 | -0.15 | -0.35 | -0.21 |  |
| L35 | -0.69 | 0.22 | -0.31 | -0.05 | -0.17 | -0.27 | -0.19 | -0.26 |
| L36 | -0.20 | 1.14 | -0.11 | 0.71 | -0.47 | 0.53 | 1.24 | 1.42 |
| L37 | 0.34 | 4.62 | -0.28 | 0.21 |  | 0.45 | 0.39 | -0.12 |
| L38 | 0.55 | 3.65 |  |  |  | 0.48 |  | 0.69 |
| L39 | -0.12 | 6.33 | -0.09 | 0.63 |  | -0.32 | -0.32 | -0.66 |
| L40 | 0.03 | 1.10 | -0.08 | 0.13 |  | -0.35 | -0.39 | -1.00 |
| L41 |  | 0.18 | -0.99 | -0.42 | -0.91 | -0.31 | -0.87 |  |
| L42 | -0.09 | 1.01 | -0.09 | 0.33 | 0.03 | -0.32 | -0.18 | 0.58 |
| L43 | -1.34 | 2.12 | -1.49 | -0.27 | -0.48 | 0.07 | -0.52 |  |
| L44 | 0.66 | 4.10 | -1.02 | 0.06 | -0.19 | -1.06 | -0.37 | -2.63 |
| L45 | 0.84 | 0.51 | -0.62 | 0.25 | -0.24 | -0.64 | 0.28 | -3.44 |
| L46 | 0.70 | 3.63 | -0.84 | 0.45 | -0.94 | -1.14 | 0.11 | -0.08 |
| L47 | 0.59 | 1.83 | -0.95 | 0.18 | -0.46 | -0.30 | 0.48 |  |
| L48 | -0.16 | -0.54 | -0.85 | -0.25 | -0.43 | -0.69 | -0.43 |  |
| L49 | 1.22 | 1.25 | -1.26 | -0.38 | -0.67 | -0.41 | -0.28 |  |
| L50 | -0.45 |  | -0.91 | -0.36 | -0.42 | -0.54 | -0.20 | -3.51 |
| L51 |  |  | -0.21 | 0.18 | 0.56 | 0.10 | 0.56 |  |
| L52 | 0.41 |  | -1.04 | 0.85 | 0.02 | -0.86 | 1.10 |  |
| L53 | -0.49 | 1.12 | -0.26 | 0.56 | 0.40 | -0.20 | 0.34 |  |
| L54 | 0.44 |  | -0.74 | -0.36 | -1.45 | -3.70 | -0.43 |  |
| L55 | -0.60 | 3.98 | -0.54 | 0.37 | 0.08 | -0.21 | 0.37 |  |
| L56 | -0.20 | 1.28 | -0.76 | 0.31 | -0.33 | -0.15 | 0.63 |  |
| L57 | 0.35 | 1.85 | -1.15 | 0.07 | -0.36 | -0.24 | 0.72 |  |
| L58 | -0.89 | 0.57 | -0.13 | -0.14 | -0.12 | -0.20 | -0.06 | -0.66 |
| L59 |  | 2.47 | 0.29 | 2.42 | 1.66 | -0.33 | 0.89 | 5.24 |
| L60 | -0.86 | -0.89 | -0.88 | -0.82 | -0.93 | -1.08 | -1.59 | -1.47 |
| L61 |  | 3.75 | -0.97 | 0.19 | -0.68 | 0.21 | 0.70 |  |
| L62 |  | 1.99 |  | 0.12 |  | -1.45 | 0.99 | 0.53 |
| L63 |  | 3.88 | 0.17 | 1.38 | 0.51 | 0.47 | 1.07 | 5.13 |
| L64 | -0.38 | -0.07 | -0.62 | -0.62 | -0.58 | -0.53 | -0.54 | -0.07 |
| L65 | -1.17 | 0.84 | -0.50 | -0.33 | -0.79 | -0.28 | -0.19 | 0.67 |
| L66 | -0.67 | 0.10 | -0.51 | -0.44 | -0.91 | -0.45 | -0.36 | -0.07 |
| L67 | -1.25 | 0.02 | -0.55 | -0.67 | -0.98 | -0.64 | -0.55 | -1.66 |
| L68 | -0.96 | -0.19 | -0.70 | -0.49 | -1.30 | -0.41 | -0.48 |  |
| L69 | -0.62 |  | -1.05 | -1.34 | -1.13 | 0.19 | 0.05 |  |
| L70 | -0.97 | -0.28 | -0.45 | -0.48 |  | -0.97 | -0.50 | 0.25 |
| L71 | -0.71 | 0.27 | -0.75 | -0.74 |  | -0.70 | -1.09 | -1.17 |
| L72 | -0.84 | 2.56 | -0.43 | -0.55 | -0.11 | -0.03 | 0.13 | -0.87 |
| L73 |  | 2.77 | 0.54 | 1.53 | 0.35 | -0.21 | 0.27 |  |
| L74 |  | 3.19 | -1.51 | -1.35 | -1.32 | 0.34 | -0.24 |  |
| L75 |  | 2.36 | 0.49 | 0.88 | 0.97 | 0.33 | 0.29 |  |
| L76 | 0.86 | 1.20 | -0.15 | 0.53 |  | -0.12 | 0.08 | 1.07 |
| L77 | 0.34 | 0.87 | -0.21 | 0.04 | 0.02 | -0.43 | -0.12 | 0.81 |
| L78 | 0.30 | 0.79 | -1.04 | 0.42 | -0.61 | 0.81 | 0.38 |  |
| L79 | -0.08 | 1.10 | -0.06 | 1.17 | 0.52 | -0.68 | 0.15 |  |
| L80 | 2.87 | -1.60 | 1.19 | 0.33 | -0.11 | -1.73 | -1.04 |  |
| L81 | -1.03 | -1.02 | 0.51 | 0.00 | -0.32 | -1.10 | -0.03 |  |
| L82 | 0.24 | 1.10 | -1.02 | -0.18 | -0.46 | 0.30 | 0.49 |  |
| L83 | -1.41 | -0.28 | -3.27 | -1.84 | -2.10 | -0.94 | -1.25 |  |
| L84 | 1.15 | -2.60 | -1.69 | -1.37 | -0.44 | -1.84 | -1.06 |  |
| L85 | -1.21 | 1.40 | -0.54 | -1.16 | -1.17 | -0.99 | -1.23 |  |
| L86 | -0.87 | 1.04 | -0.17 | -0.02 | -0.08 | -1.34 | -0.78 |  |
| L87 | 0.85 | 2.77 | -0.66 | -0.60 | 0.98 | -0.10 | -0.79 |  |
| L88 | -0.02 | 1.15 | -1.49 | -0.91 | 0.29 | -1.10 | -1.18 |  |
| L89 | 2.38 | 0.98 | -1.96 | -1.49 | -2.00 | -1.37 | -1.37 |  |
| L90 | 3.97 | 2.42 | -1.57 | -1.35 | -1.20 | -1.45 | -1.24 |  |
| L91 | 0.11 | -2.02 | -0.20 | -1.20 | -2.13 | -1.74 | -1.83 |  |
| L92 | 0.85 | 1.10 | -2.12 | -1.75 | -1.08 | -0.74 | -1.38 |  |
| L93 | -0.12 | 2.30 | -0.85 | -0.79 | -0.54 | -1.24 | -0.75 |  |
| L94 |  | 0.10 | -0.64 | 0.36 | -0.59 | 0.13 | 0.36 |  |
| L95 | 0.71 | -0.60 | -0.77 | -0.06 | -0.45 | -1.01 | -0.83 |  |
| L96 |  | 2.33 | -0.26 | 0.67 | 0.11 | 0.90 | 0.65 |  |
| L97 |  | 0.48 | -1.58 | -0.81 | -1.07 | -0.80 | -0.95 |  |
| L98 |  | -1.17 | -1.01 | -1.51 | -1.29 | -0.69 | -1.46 |  |
| L99 |  | -0.36 | -0.29 | -0.43 | -0.39 | -0.58 | -0.63 |  |
| L100 |  | -0.36 | -0.67 | -0.89 | -0.54 | -0.80 | -1.01 |  |
| L101 | -0.11 | -0.08 | -0.52 | -0.01 | -0.50 | 0.44 | 0.15 | 0.23 |
| L102 | 0.21 | 2.52 | 0.11 | 0.27 | -0.22 | 0.11 | 0.61 | 1.46 |
| L103 | -0.04 | 1.75 | 0.09 | -0.14 | -0.29 | -0.36 | -0.30 | 0.97 |
| L104 |  | 1.61 | 0.28 | 0.14 | -0.20 | -0.56 | -0.06 |  |
| L105 |  | 0.90 | 0.72 | 0.89 | 0.69 | -1.05 | 0.14 |  |
| L106 |  | 1.01 | 0.01 | -0.23 | -0.23 | -0.93 | -0.18 |  |
| L107 | -1.58 | 0.58 | 0.16 | -0.22 | -0.68 | -0.81 | -0.44 | -0.80 |
| L108 | -1.26 | 0.88 | -0.19 | -0.02 | -0.62 | 0.36 | -0.15 | 0.34 |
| L109 | 0.36 | 2.03 | -0.21 | -0.36 |  |  | -0.26 | 3.54 |
| L110 | -0.76 | 3.24 | -0.63 | 0.11 |  | 0.28 |  | -2.10 |

**Table S6 $E_{r}^{i}$ and RI values of heavy metals in sediments from different lakes in China.**

| Lake | $E_{r}^{i}$ | | | | | | | | RI |
| --- | --- | --- | --- | --- | --- | --- | --- | --- | --- |
|  | As | Cd | Cr | Cu | Ni | Pb | Zn | Hg |  |
| L1 | 22.4 | 133.0 | 1.7 | 6.4 | 2.2 | 9.4 | 2.5 | 290.9 | 468.5 |
| L2 |  | 114.4 | 2.6 | 12.4 | 2.9 | 8.3 | 1.9 |  | 142.6 |
| L3 | 9.4 | 68.0 |  | 8.8 | 2.4 | 5.1 | 2.6 | 48.5 | 144.8 |
| L4 |  | 151.5 | 2.8 | 12.3 | 3.3 | 8.2 | 1.9 |  | 180.1 |
| L5 |  | 117.5 | 2.6 | 8.9 | 2.7 | 8.0 | 1.6 |  | 141.3 |
| L6 |  | 154.6 | 2.9 | 12.0 | 3.4 | 9.1 | 2.1 |  | 184.2 |
| L7 |  | 102.1 | 2.5 | 8.6 | 2.9 | 7.7 | 1.7 |  | 125.4 |
| L8 |  | 71.1 | 2.6 | 10.2 | 3.4 | 6.4 | 1.7 |  | 95.4 |
| L9 |  | 68.0 | 2.0 | 6.4 | 2.1 | 5.4 | 1.2 |  | 85.2 |
| L10 |  | 108.2 | 2.6 | 10.2 | 3.1 | 7.2 | 1.9 |  | 133.2 |
| L11 |  | 262.9 | 2.3 | 11.2 | 2.9 | 10.6 | 2.1 |  | 292.0 |
| L12 |  | 120.6 | 2.7 | 9.3 | 3.2 | 9.2 | 1.9 |  | 147.0 |
| L13 |  | 80.4 | 2.6 | 9.4 | 2.9 | 6.7 | 1.7 |  | 103.7 |
| L14 |  | 92.8 | 2.1 | 8.6 | 2.7 | 5.9 | 1.5 |  | 113.5 |
| L15 |  | 136.1 | 2.6 | 11.4 | 3.1 | 8.3 | 1.9 |  | 163.4 |
| L16 |  |  | 3.1 | 2.5 |  | 5.1 | 1.5 |  | 12.2 |
| L17 |  | 770.3 | 2.0 |  |  | 5.6 |  | 34.8 | 812.7 |
| L18 |  | 85.1 | 1.9 | 6.7 | 2.7 | 6.6 | 1.0 |  | 104.1 |
| L19 |  | 380.4 | 2.0 | 8.4 | 3.1 | 10.9 | 7.3 | 92.3 | 504.3 |
| L20 |  | 589.3 | 2.2 | 10.0 | 3.2 | 17.8 | 14.7 | 87.2 | 724.2 |
| L21 |  | 203.6 | 9.2 | 32.4 | 34.5 | 2.3 | 1.6 | 348.7 | 632.4 |
| L22 |  | 43.2 | 1.4 | 16.0 |  | 5.7 | 1.9 |  | 68.2 |
| L23 | 14.9 | 35.1 | 1.8 | 14.4 | 9.7 | 5.1 | 1.4 | 240.0 | 322.3 |
| L24 |  | 55.8 | 3.1 | 8.1 | 3.4 | 5.1 | 1.2 |  | 76.7 |
| L25 |  | 48.8 | 2.6 | 4.9 | 2.2 | 4.5 | 0.9 |  | 63.9 |
| L26 | 10.0 | 90.7 | 3.1 | 8.1 | 4.2 | 6.0 | 1.8 | 118.9 | 242.7 |
| L27 | 6.3 | 17.4 | 0.6 | 1.5 | 0.6 | 3.9 | 0.3 | 10.8 | 41.5 |
| L28 | 6.8 | 20.9 | 1.0 | 2.4 | 1.1 | 4.2 | 0.5 | 10.8 | 47.7 |
| L29 | 1.5 | 2198.9 | 2.0 | 8.2 | 1.9 | 13.0 | 1.2 |  | 2226.6 |
| L30 | 13.7 | 6.4 | 1.6 | 6.0 |  | 4.8 | 0.8 | 100.0 | 133.2 |
| L31 |  | 166.2 | 2.4 | 7.6 | 2.7 | 7.0 | 1.9 |  | 187.8 |
| L32 | 8.6 | 40.5 | 1.6 | 4.8 | 1.8 | 4.6 | 0.8 | 11.8 | 74.5 |
| L33 | 28.5 | 139.5 | 2.7 | 8.1 | 2.8 | 5.4 | 1.5 | 40.0 | 228.6 |
| L34 |  |  | 2.0 | 6.6 | 2.7 | 5.9 | 1.3 |  | 18.5 |
| L35 | 9.3 | 52.3 | 2.4 | 7.2 | 2.7 | 6.2 | 1.3 | 35.0 | 116.4 |
| L36 | 13.0 | 99.4 | 2.8 | 12.3 | 2.2 | 10.8 | 3.6 | 160.0 | 304.1 |
| L37 | 18.9 | 1107.1 | 2.5 | 8.7 |  | 10.3 | 2.0 | 55.2 | 1204.6 |
| L38 | 22.0 | 566.7 |  |  |  | 10.4 |  | 96.6 | 695.6 |
| L39 | 13.8 | 3619.0 | 2.8 | 11.6 |  | 6.0 | 1.2 | 37.9 | 3692.4 |
| L40 | 15.3 | 96.2 | 2.8 | 8.2 |  | 5.9 | 1.1 | 30.0 | 159.6 |
| L41 |  | 50.9 | 1.5 | 5.6 | 1.6 | 6.1 | 0.8 |  | 66.6 |
| L42 | 14.1 | 90.6 | 2.8 | 9.4 | 3.1 | 6.0 | 1.3 | 90.0 | 217.3 |
| L43 | 5.9 | 195.2 | 1.1 | 6.2 | 2.2 | 7.9 | 1.0 |  | 219.5 |
| L44 | 23.7 | 771.4 | 1.5 | 7.8 | 2.6 | 3.6 | 1.2 | 9.7 | 821.5 |
| L45 | 26.9 | 64.3 | 2.0 | 8.9 | 2.5 | 4.8 | 1.8 | 5.5 | 116.7 |
| L46 | 24.4 | 557.1 | 1.7 | 10.3 | 1.6 | 3.4 | 1.6 | 56.7 | 656.8 |
| L47 | 22.7 | 159.5 | 1.5 | 8.5 | 2.2 | 6.1 | 2.1 |  | 202.6 |
| L48 | 13.4 | 31.0 | 1.7 | 6.3 | 2.2 | 4.6 | 1.1 |  | 60.3 |
| L49 | 34.9 | 107.1 | 1.3 | 5.7 | 1.9 | 5.6 | 1.2 |  | 157.8 |
| L50 | 11.0 |  | 1.6 | 5.8 | 2.2 | 5.2 | 1.3 | 5.3 | 32.4 |
| L51 |  |  | 2.6 | 8.5 | 4.4 | 8.0 | 2.2 |  | 25.8 |
| L52 | 19.9 |  | 1.5 | 13.5 | 3.0 | 4.1 | 3.2 |  | 45.2 |
| L53 | 10.7 | 97.6 | 2.5 | 11.0 | 3.9 | 6.5 | 1.9 |  | 134.2 |
| L54 | 20.4 |  | 1.8 | 5.8 | 1.1 | 0.6 | 1.1 |  | 30.8 |
| L55 | 9.9 | 709.5 | 2.1 | 9.7 | 3.2 | 6.5 | 1.9 |  | 742.7 |
| L56 | 13.1 | 109.5 | 1.8 | 9.3 | 2.4 | 6.8 | 2.3 |  | 145.2 |
| L57 | 19.2 | 161.9 | 1.4 | 7.9 | 2.3 | 6.3 | 2.5 |  | 201.5 |
| L58 | 8.1 | 66.7 | 2.7 | 6.8 | 2.8 | 6.5 | 1.4 | 38.1 | 133.2 |
| L59 |  | 248.5 | 3.7 | 40.1 | 9.5 | 6.0 | 2.8 | 2270.3 | 2580.8 |
| L60 | 8.3 | 24.2 | 1.6 | 4.2 | 1.6 | 3.5 | 0.5 | 21.6 | 65.6 |
| L61 |  | 606.1 | 1.5 | 8.6 | 1.9 | 8.7 | 2.4 |  | 629.1 |
| L62 |  | 178.8 |  | 8.1 |  | 2.7 | 3.0 | 86.5 | 279.1 |
| L63 |  | 661.1 | 3.4 | 19.5 | 4.3 | 10.4 | 3.2 | 2108.1 | 2809.8 |
| L64 | 11.5 | 42.9 | 2.0 | 4.9 | 2.0 | 5.2 | 1.0 | 57.1 | 126.6 |
| L65 | 6.6 | 80.4 | 2.1 | 6.0 | 1.7 | 6.2 | 1.3 | 95.2 | 199.6 |
| L66 | 9.4 | 48.2 | 2.1 | 5.5 | 1.6 | 5.5 | 1.2 | 57.1 | 130.7 |
| L67 | 6.3 | 45.5 | 2.1 | 4.7 | 1.5 | 4.8 | 1.0 | 19.0 | 85.0 |
| L68 | 7.7 | 39.4 | 1.8 | 5.3 | 1.2 | 5.6 | 1.1 |  | 62.3 |
| L69 | 9.8 |  | 1.4 | 3.0 | 1.4 | 8.6 | 1.6 |  | 25.7 |
| L70 | 7.7 | 37.0 | 2.2 | 5.4 |  | 3.8 | 1.1 | 71.6 | 128.7 |
| L71 | 9.2 | 54.3 | 1.8 | 4.5 |  | 4.6 | 0.7 | 26.7 | 101.7 |
| L72 | 8.4 | 265.8 | 2.2 | 5.1 | 2.8 | 7.4 | 1.6 | 32.8 | 326.1 |
| L73 |  | 307.6 | 4.4 | 21.7 | 3.8 | 6.5 | 1.8 |  | 345.8 |
| L74 |  | 410.1 | 1.1 | 2.9 | 1.2 | 9.5 | 1.3 |  | 426.1 |
| L75 |  | 231.6 | 4.2 | 13.8 | 5.9 | 9.4 | 1.8 |  | 266.8 |
| L76 | 27.2 | 103.6 | 2.7 | 10.8 |  | 6.9 | 1.6 | 126.3 | 279.1 |
| L77 | 19.0 | 82.1 | 2.6 | 7.7 | 3.0 | 5.6 | 1.4 | 105.3 | 226.7 |
| L78 | 18.5 | 77.8 | 1.5 | 10.0 | 2.0 | 13.2 | 1.9 |  | 124.9 |
| L79 | 14.2 | 96.3 | 2.9 | 16.9 | 4.3 | 4.7 | 1.7 |  | 140.9 |
| L80 | 109.6 | 14.8 | 6.8 | 9.5 | 2.8 | 2.3 | 0.7 |  | 146.5 |
| L81 | 7.4 | 22.2 | 4.3 | 7.5 | 2.4 | 3.5 | 1.5 |  | 48.8 |
| L82 | 17.7 | 96.3 | 1.5 | 6.6 | 2.2 | 9.2 | 2.1 |  | 135.5 |
| L83 | 5.6 | 37.0 | 0.3 | 2.1 | 0.7 | 3.9 | 0.6 |  | 50.3 |
| L84 | 33.4 | 7.4 | 0.9 | 2.9 | 2.2 | 2.1 | 0.7 |  | 49.7 |
| L85 | 6.5 | 118.5 | 2.1 | 3.4 | 1.3 | 3.8 | 0.6 |  | 136.2 |
| L86 | 8.2 | 92.6 | 2.7 | 7.4 | 2.8 | 3.0 | 0.9 |  | 117.6 |
| L87 | 27.1 | 307.4 | 1.9 | 4.9 | 5.9 | 7.0 | 0.9 |  | 355.1 |
| L88 | 14.8 | 100.0 | 1.1 | 4.0 | 3.7 | 3.5 | 0.7 |  | 127.6 |
| L89 | 78.1 | 88.9 | 0.8 | 2.7 | 0.8 | 2.9 | 0.6 |  | 174.6 |
| L90 | 235.0 | 240.7 | 1.0 | 2.9 | 1.3 | 2.7 | 0.6 |  | 484.4 |
| L91 | 16.1 | 11.1 | 2.6 | 3.3 | 0.7 | 2.3 | 0.4 |  | 36.5 |
| L92 | 27.0 | 96.3 | 0.7 | 2.2 | 1.4 | 4.5 | 0.6 |  | 132.7 |
| L93 | 13.8 | 222.2 | 1.7 | 4.3 | 2.1 | 3.2 | 0.9 |  | 248.1 |
| L94 |  | 48.1 | 1.9 | 9.7 | 2.0 | 8.2 | 1.9 |  | 71.8 |
| L95 | 24.5 | 29.6 | 1.8 | 7.2 | 2.2 | 3.7 | 0.8 |  | 69.8 |
| L96 |  | 225.9 | 2.5 | 12.0 | 3.2 | 14.0 | 2.4 |  | 260.0 |
| L97 |  | 63.0 | 1.0 | 4.3 | 1.4 | 4.3 | 0.8 |  | 74.8 |
| L98 |  | 20.0 | 1.5 | 2.6 | 1.2 | 4.7 | 0.5 |  | 30.6 |
| L99 |  | 35.0 | 2.5 | 5.6 | 2.3 | 5.0 | 1.0 |  | 51.3 |
| L100 |  | 35.0 | 1.9 | 4.1 | 2.1 | 4.3 | 0.7 |  | 48.0 |
| L101 | 13.9 | 42.5 | 2.1 | 7.5 | 2.1 | 10.2 | 1.7 | 70.6 | 150.5 |
| L102 | 17.3 | 257.3 | 3.2 | 9.0 | 2.6 | 8.1 | 2.3 | 165.5 | 465.4 |
| L103 | 14.6 | 151.4 | 3.2 | 6.8 | 2.5 | 5.8 | 1.2 | 117.2 | 302.7 |
| L104 |  | 137.6 | 3.6 | 8.3 | 2.6 | 5.1 | 1.4 |  | 158.7 |
| L105 |  | 83.9 | 5.0 | 13.9 | 4.8 | 3.6 | 1.6 |  | 112.9 |
| L106 |  | 90.8 | 3.0 | 6.4 | 2.6 | 3.9 | 1.3 |  | 108.1 |
| L107 | 5.0 | 67.4 | 3.3 | 6.4 | 1.9 | 4.3 | 1.1 | 34.5 | 124.0 |
| L108 | 6.3 | 82.6 | 2.6 | 7.4 | 2.0 | 9.6 | 1.3 | 75.9 | 187.6 |
| L109 | 19.3 | 184.3 | 2.6 | 5.9 |  |  | 1.3 | 697.7 | 911.0 |
| L110 | 8.8 | 424.3 | 1.9 | 8.1 |  | 9.1 |  | 14.0 | 466.2 |

**Table S7 The proportion of grades of potential ecological risk in whole lake from China.**

| The grade of ecological risk | Low risk | Moderate risk | High risk | Very high risk |
| --- | --- | --- | --- | --- |
| % of lake sample in each grade | 54.6% | 21.8% | 10.9% | 12.7% |
